# Genome-wide association for grain morphology in synthetic hexaploid wheats using digital imaging analysis

**DOI:** 10.1186/1471-2229-14-128

**Published:** 2014-05-09

**Authors:** Awais Rasheed, Xianchun Xia, Francis Ogbonnaya, Tariq Mahmood, Zongwen Zhang, Abdul Mujeeb-Kazi, Zhonghu He

**Affiliations:** 1 Institute of Crop Science, National Wheat Improvement Center, Chinese Academy of Agricultural Sciences (CAAS), 12 Zhongguancun South Street, Beijing 100081, China; 2 Grain Research and Development Corporation (GRDC), Barton, ACT 2600, Australia; 3 Department of Plant Sciences, Quaid-i-Azam University, Islamabad 45320, Pakistan; 4 Bioversity International c/o CAAS, 12 Zhongguancun South Street, Beijing 100081, China; 5 National Institute of Biotechnology and Genetic Engineering (NIBGE), Faisalabad, Pakistan; 6 International Maize and Wheat Improvement Center (CIMMYT) China Office, c/o CAAS, 12 Zhongguancun South Street, Beijing 100081, China

## Abstract

**Background:**

Grain size and shape greatly influence grain weight which ultimately enhances grain yield in wheat. Digital imaging (DI) based phenomic characterization can capture the three dimensional variation in grain size and shape than has hitherto been possible. In this study, we report the results from using digital imaging of grain size and shape to understand the relationship among different components of this trait, their contribution to enhance grain weight, and to identify genomic regions (QTLs) controlling grain morphology using genome wide association mapping with high density diversity array technology (DArT) and allele-specific markers.

**Results:**

Significant positive correlations were observed between grain weight and grain size measurements such as grain length (*r* = 0.43), width, thickness (*r* = 0.64) and factor from density (FFD) (*r* = 0.69). A total of 231 synthetic hexaploid wheats (SHWs) were grouped into five different sub-clusters by Bayesian structure analysis using unlinked DArT markers. Linkage disequilibrium (LD) decay was observed among DArT loci > 10 cM distance and approximately 28% marker pairs were in significant LD. In total, 197 loci over 60 chromosomal regions and 79 loci over 31 chromosomal regions were associated with grain morphology by genome wide analysis using general linear model (GLM) and mixed linear model (MLM) approaches, respectively. They were mainly distributed on homoeologous group 2, 3, 6 and 7 chromosomes. Twenty eight marker-trait associations (MTAs) on the D genome chromosomes 2D, 3D and 6D may carry novel alleles with potential to enhance grain weight due to the use of untapped wild accessions of *Aegilops tauschii*. Statistical simulations showed that favorable alleles for thousand kernel weight (TKW), grain length, width and thickness have additive genetic effects. Allelic variations for known genes controlling grain size and weight, viz. *TaCwi-2A*, *TaSus-2B*, *TaCKX6-3D* and *TaGw2-6A,* were also associated with TKW, grain width and thickness. *In silico* functional analysis predicted a range of biological functions for 32 DArT loci and receptor like kinase, known to affect plant development, appeared to be common protein family encoded by several loci responsible for grain size and shape.

**Conclusion:**

Conclusively, we demonstrated the application and integration of multiple approaches including high throughput phenotyping using DI, genome wide association studies (GWAS) and *in silico* functional analysis of candidate loci to analyze target traits, and identify candidate genomic regions underlying these traits. These approaches provided great opportunity to understand the breeding value of SHWs for improving grain weight and enhanced our deep understanding on molecular genetics of grain weight in wheat.

## Background

Bread wheat (*Triticum aestivum* L.) is one of the most important crops providing food to more than 4.5 billion people in 94 developing countries [1]. It is a huge challenge to ensure global food security through sustainable wheat production for the projected population with the increasing adverse impact of climate change [2]. More scientific and targeted exploitation of wild crop relatives is considered to be a valuable strategy to deal with this challenge [3]. *Aegilops tauschii*, D-genome donor to bread wheat, and their derived SHWs are major reservoir of favorable alleles for economic traits and have been considered as prioritized genetic resources for wheat genetic improvement [4]. Significant variations have been reported in SHWs including grain weight [5,6], bread-making quality [7], nutritional quality [8], resistance to biotic stresses [9] and abiotic stresses [4,10]. While previous use of SHWs focused on their mining for biotic stresses, there is increasing focus on its potential to contribute favorable genes for grain yield as demonstrated by several SHWs derived varieties released in China, Spain, Ecuador and Mexico [4].

Grain yield in wheat is the most important agronomic trait. It is underpinned by two numerical components i.e., grain weight and grains per m^2^. In the past four decades, improvement of grain yield has come from increased grains per m^2^ or larger grain sizes, due to the utilization of *Rht* genes in wheat breeding [11]. Improvement of the TKW is considered to be an important approach for further improving yield potential in Yellow and Huai valleys in China and Northwest Mexico [12]. SHWs exhibited significant variation for grain weight compared to bread wheat and TKW of up to 67 g have been reported in Mexico [11]. Cooper et al. [13,14] performed two consecutive experiments over two years to examine the yield potential of SHWs under rain-fed field conditions and concluded that grain weight is the most heritable trait and even some lines with higher number of spikes and higher number of grains per spike maintained their grain size and weight.

Grain size and shape in wheat significantly affect grain weight and flour yield [15] and appear to be breeding target dictated by market and industry requirements [16]. Theoretical models predict that milling yield could be increased by optimizing grain shape and size with large and spherical grains being the optimum grain morphology [17]. However, accurate characterization of grain size and shape remains a big challenge due to laborious, time consuming techniques and complex nature of wheat grain shape. Recent advances in the photometric techniques provide more concise, potentially cheaper phenotypic information and can better devolve the function of complex traits into individual genetic components [18]. DI analysis is proving to be a useful tool and can capture the three dimensional shapes of grains using different image orientations [15,19].

Discovery of QTLs for grain weight and their validation are important steps to accelerate the speed of successful deployment of favorable alleles through marker-assisted selection [20]. The relative advantages of association mapping (AM) or linkage disequilibrium (LD) mapping over the linkage mapping for the underlying trait mechanisms have been reported [21]. In wheat, several reports have described the identification of QTLs for grain size and weight [22-31]. However, only few studies targeted QTLs for grain shape [15,16,19], and only Gegas et al. [16] reported these function in wild species of wheat relatives. Further, the development of functional markers and cloning of genes relevant to grain weight have become major research focus in the past few years. Many QTLs for grain size and weight in rice have been fine-mapped and cloned in wheat including *TaCwi-1A *[32], *TaSus2-2B *[33], *TaGw2-6A *[34], *TaCKX6-D1 *[35], *TaSap1-A1 *[36], *TaGS1-6D *[37] and *TaLsu1 *[38].

The objectives of current study were i) to characterize SHWs genotypes for grain size and shape and determine its relationship with grain weight using high-throughput digital imaging phenotyping, ii) to identify the potential genomic regions underlying grain phenotypes using DArT markers by genome wide association analysis, and iii) to investigate potential function of QTLs identified using sequences of DArT markers significantly associated with grain phenotypes.

## Results

### Variation in grain morphology of synthetic hexaploid wheat

Phenotypic data for grain morphology descriptors were averaged from two cropping seasons in 2010–2011 and 2011–2012. The basic statistics for grain size and shape traits observed in SHWs are given as Additional file 1: Table S2 and frequency distributions for these traits are shown as Additional file 2: Figure S1. Broad sense heritability was found to be moderate to high for the 29 traits and ranged between 0.65 and 0.92 for vertical principal component-2 (VPC2) and vertical area (VArea), respectively. Seventeen SHWs showed mean TKW over 60 g and were mostly derived from different durum and *Ae. tauschii* accessions. Maximum TKW (64.3 g) was observed in AUS34448 and minimum (36.1 g) in AUS30288. Maximum numbers of SHWs (24) were derived from durum wheat variety Croc_1 which exhibited greater variation for TKW that ranged between 37.1 to 61.4 g. Similar trend was observed for other measurements including grain width, length and thickness. Some direct measurements such as grain length, width, thickness and indirect measurements like factor from density (FFD) and volume are considered to be very important for determining grain size, shape and weight. Grain length ranged from 6.8 mm (AUS33405) to 9.3 mm (AUS34240) with an average of 8.2 mm. Similarly, grain width ranged from 2.8 mm (AUS30288) to 3.8 mm (AUS34239) with an average of 3.3 mm. Similar trend of variability was found for horizontal area and vertical area of grain which are derivatives of horizontal and vertical major and minor axis, respectively. Grain volume ranged from 25 mm^3^ (AUS30632) to 51 mm^3^ (AUS34239) with an average of 37.8 mm^3^. The other very important derived measurement FFD ranged from 3.2 (AUS30300) to 5.71 (AUS30283) with an average of 4.74.

### Pearsons’s correlation and path coefficient analysis for grain morphology traits

**Figure 1:**
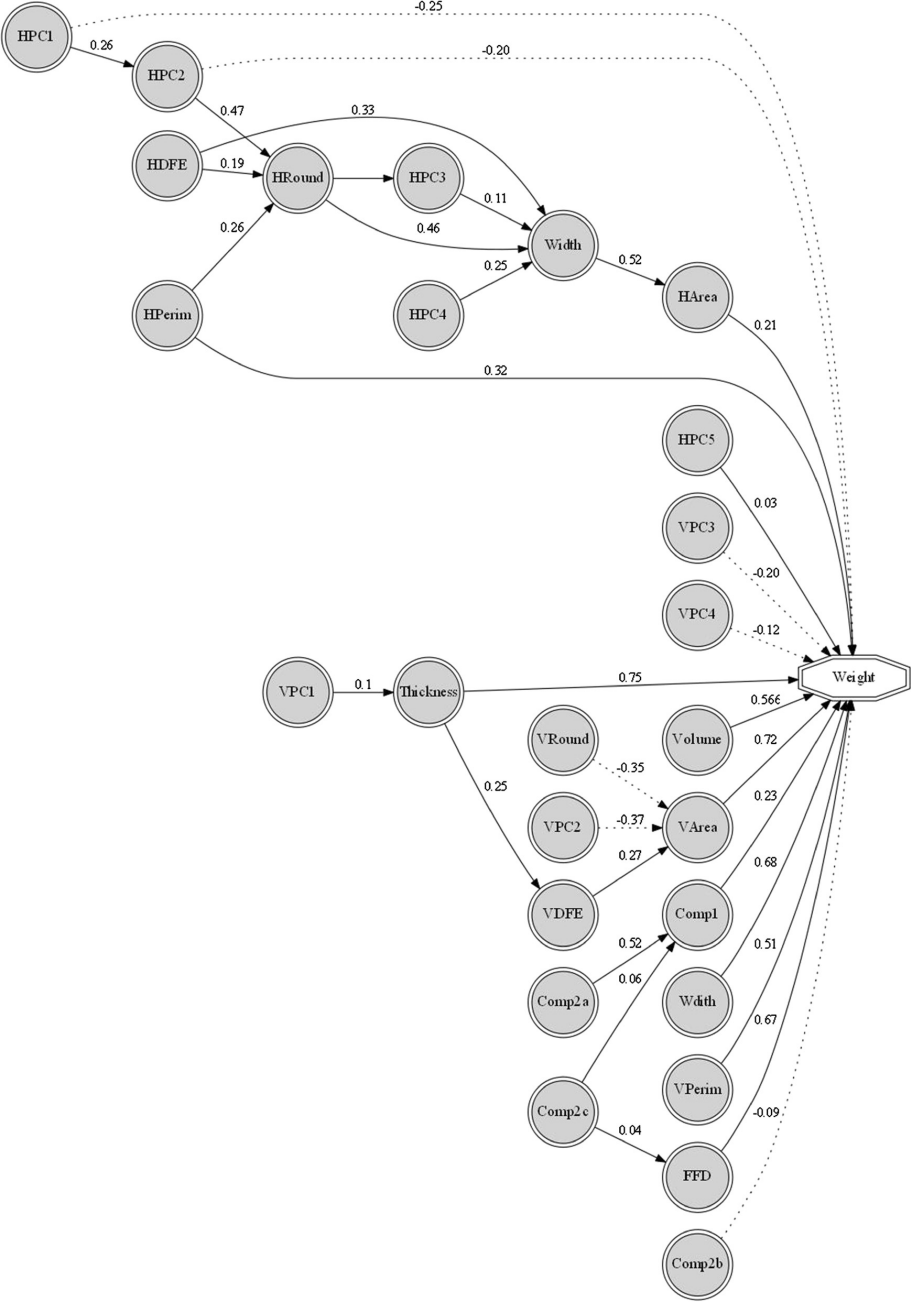
**Path analysis for direct and indirect effects of seed size and shape descriptors to grain weight.** Dotted lines represent the negative effects of the descriptor on grain weight.

Perason’s coefficient of correlation was calculated for all traits based on the data averaged from two seasons (Table 1). The maximum positive correlation (0.84) was observed between grain volume and horizontal deviation from ellipse (HDFE), followed by *r* = 0.81 between horizontal area and composite 1 (Comp1). The maximum negative correlation (-0.76) was observed between vertical roundness (VRound) and vertical principal component 3 (VPC3). The co-efficient of correlation between grain size direct measurements and grain weight was almost positive and significant. For example, grain length and grain width had positive correlation with TKW with estimate of *r* = 0.43 and *r* = 0.64, respectively. Similarly, grain thickness is highly correlated with TKW (*r* = 076). The important derived measurements like volume and FFD were also positively correlated with TKW, with *r* = 0.78 and *r* = 0.69, respectively. Grain volume has higher value of correlation with vertical area (*r* = 0.80) as compared to horizontal area (*r* = 0.58). Similarly, vertical and horizontal principal components have non-significant mixed trend of correlation with grain weight and therefore not shown in Table 1.In order to have a clear understanding of the effect of individual measurement on grain weight, path co-efficient analysis was computed by taking TKW as dependent variable. Due to the higher number (29) of variables of grain size and shape, all the descriptors were partitioned into three groups. First and second groups consisted of ten variables describing horizontal and vertical aspects of grain size and shapes, respectively. The third group consisted of nine variables and described some miscellaneous derivative measurements. A pictorial representation of path analysis of the three descriptor groups is given in Figure 1. Grain thickness exhibited maximum direct effect on grain weight followed by VArea, while horizontal area (HArea) has relatively less direct effect on grain weight. Some principal components like HPC1, HPC2, VPC3 and VPC4 showed direct negative effect on grain weight. Both of the important derivatives such as grain volume and FFD have direct positive effect on grain weight. Horizontal and vertical deviations from the ellipse have indirect positive effect on grain weight and both vertical and horizontal perimeters have direct positive effect on grain weight because these are derivatives of grain length, width and thickness.

**Table 1:** Pearson’s co-efficient of correlation for important grain size and shape descriptots in D genome synthetic hexaploid wheats

**Variables**	**HArea**	**HPerim.**	**Length**	**HRound**	**HDFE**	**Aspect ratio**	**VArea**	**VPerim.**	**Width**	**Thickness**	**VRound**	**VDFE**	**Volume**	**FFD**
HPerim	0.51**													
Length	0.73**	0.75**												
HRound	0.06	-0.52**	-0.64**											
HDFE	0.92**	0.68**	0.92**	-0.31*										
Aspect ratio	-0.10	0.51**	0.6**	-0.98**	0.28*									
VArea	0.58**	0.44*	0.3*	0.22*	0.47**	-0.24*								
VPerim	0.49**	0.47**	0.34*	0.05	0.44*	-0.06	0.71**							
Width	0.56**	0.46**	0.31*	0.19*	0.47**	-0.20*	0.96**	0.71**						
Thickness	0.55**	0.39*	0.27*	0.23*	0.43*	-0.26*	0.96**	0.66**	0.86**					
VRound	0.02	-0.09	-0.06	0.09	-0.04	-0.12	0.09**	-0.03	-0.19*	0.35**				
VDFE	0.58**	0.45*	0.31*	0.22*	0.48**	-0.23*	0.95**	0.71**	0.97**	0.95**	0.04			
Volume	0.94**	0.52**	0.63**	0.14*	0.84**	-0.17*	0.8**	0.61**	0.75**	0.8**	0.15*	0.8**		
FFD	-0.09	0.13*	-0.13*	0.09	-0.13	-0.11*	0.41*	0.22*	0.32*	0.48**	0.33*	0.4*	0.12	
TKW	0.66**	0.47**	0.43*	0.11	0.58**	-0.14*	0.72**	0.52**	0.64**	0.76**	0.26*	0.72**	0.77**	0.69**

*and **Represents significance at P < 0.05 and P < 0.01, respectively.

*HArea*: Horizontal area; *HPerim*: Horizontal perimeter; *HRound*: Horizontal roundness; *HDFE*: Horizontal deviation from ellipse; *VArea*: Verticalarea; *VPerim*: Vertical perimeter; *VRound*: Vertical roundness; *VDFE*: Vertical deviation from ellipse; *FFD*: Factor from density.

### Marker coverage and polymorphism in synthetic hexaploid wheats

The 231 SHWs were genotyped with DArT markers which are bi-allelic markers. A consensus genetic map of DArT markers based on more than 100 mapping populations was used to allocate the chromosomal position [39]. In total, 834 polymorphic DArT markers were used for final genetic and association analysis. The marker density in this population was 40 markers per chromosome. DArT markers integrated into the framework genetic map covered a total genetic distance of 2,607 cM, with an average density of one marker per 3.12 cM. The number of markers per chromosome ranged between 8 (chromosomes 5D and 5A) and 102 (chromosome 3B). However, the marker density for D-genome chromosomes was very low (20.28 per chromosome) as compared to A and B genomes. Polymorphic information content (PIC) value ranged from 0.06 to 0.499 with an average of 0.39.

### Population structure

**Figure 2:**
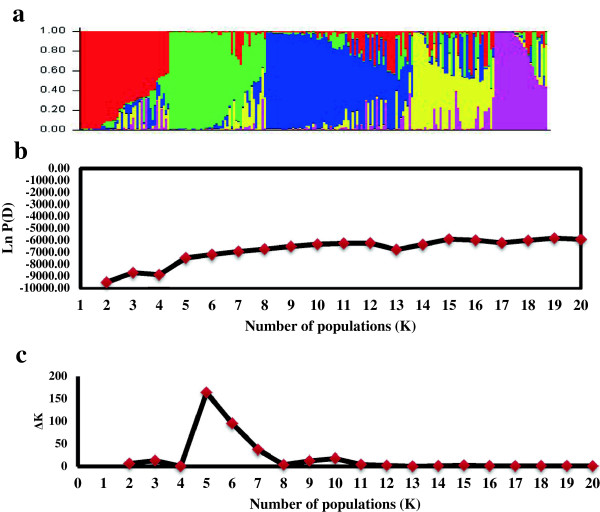
**Population structure of synthetic hexaploids based on DArT markers. a)** Membership co-efficient (Q value) where each horizontal line represents one wheat line, and partitioned into five sub-populations. **b)** Plot of the average logarithm of the probability of data likelihood (LnP (D)), as a function of the number of assumed subgroups (K), with K allowed to range from 2 to 20. **c)** Plot of the average logarithm of the probability of data delta K (ΔK), as second rate of change of the number of assumed subgroups (K), with K allowed to range from 2 to 20.

Analysis of population structure showed that the logarithm of the data likelihood (Ln P (D)) on average continued to increase with increasing numbers of assumed subpopulations (K) from 2 to 20 with exception of the depression at K4, K13 and K17 (Figure 2b). Differences between Ln P (D) values at two successive K values became non-significant after K = 5. The ad-hoc quantity based on the second order rate of change in the log probability (ΔK) showed a clear peak at K = 5 (Figure 2c), which confirmed that a K value of 5 was the most probable prediction for the number of subpopulations. The number of SHWs in the five subpopulations ranged from 27 to 67 genotypes. Maximum numbers of SHWs were observed in K3 (67) and minimum were observed in K5 (27). The average distance between sub-populations ranged from 0.08 to 0.26.

### Linkage disequilibrium patterns in germplasm panel

**Figure 3:**
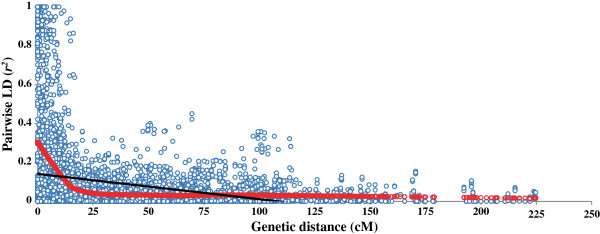
**Scatterplot of the LD statistic *r***^***2 ***^**as a function of genetic distance (cM) between pairs of DArT markers in SHWs.** The locally weighted polynomial regression-based (LOESS) representing decay of *r*^*2*^ along genetic distance is illustrated for each genome. LD critical threshold estimated from LD distribution of pairs of unlinked DArT markers is indicated by the dashed horizontal line.

LD was estimated by *r*^*2*^ at *P* ≤ 0.001 from all pairs of the DArT markers. LD patterns along 21 wheat chromosomes can be visualized as heatmaps (Additional file 3: Figure S5). On a genome-wide level, almost 58.1% of all pairs of loci were in significant LD (Table 2). The average *r*^*2*^ of genome-wide LD was 0.09. DArT markers assigned to their map position were further used to estimate inter- and intra-chromosomal LD. About 28% of inter-chromosomal pairs of loci were in significant LD, with an average *r*^*2*^ of 0.09, while 42% of intra-chromosomal pairs of loci were insignificant LD with an average *r*^*2*^ of 0.3. The extent and distribution of LD were graphically displayed by plotting intra-chromosomal *r*^*2*^ values for loci in significant LD at *P* ≤ 0.001 against the genetic distance in centi-Morgans and a second-degree LOESS curve was fitted (Figure 3). The critical value for significance of *r*^*2*^ was estimated at 0.2 according to [40], and thus all values of *r*^*2*^ > 0.2 were estimated to be due to genetic linkage. The baseline intersection with the LOESS curve was at 9 cM, which was considered as the estimate of the extent of LD in the SHW population, although in a few cases high levels of LD were observed over longer distances (*r*^*2*^ = 1 at a genetic distance of 167 cM). LD decays to an average *r*^*2*^ of 0.069 from 0.246 as the genetic distance increased to > 10 cM and the markers in complete LD also reduced to 1 from 238 (Table 2). Thus the map coverage of 6 cM was deemed appropriate to perform a genome-wide association analysis on the SHWs population.

**Table 2:** An overview of LD among whole panel of SHWs

**Classes**	**Total pairs**	**Significant (%)**	**Significant pairs**	**Mean *r***^***2***^	**Pairs in complete LD**	**Pairs (%) in LD > 0.2**	**Mean of *r***^***2***^ **> 0.2**
0-10 cM	3078	74.46	2292	0.246	238	32.78	0.348
11-20 cM	1974	59.98	1184	0.069	7	8.31	0.4
21-50 cM	2979	53.24	1586	0.049	1	3.32	0.081
>50 cM	5422	50.87	2758	0.035	1	2.16	0.29
Total	13453	58.13	7820	0.09	247	10.24	0.57

### Marker-trait associations for grain morphology in synthetic hexaploid wheats

**Figure 4:**
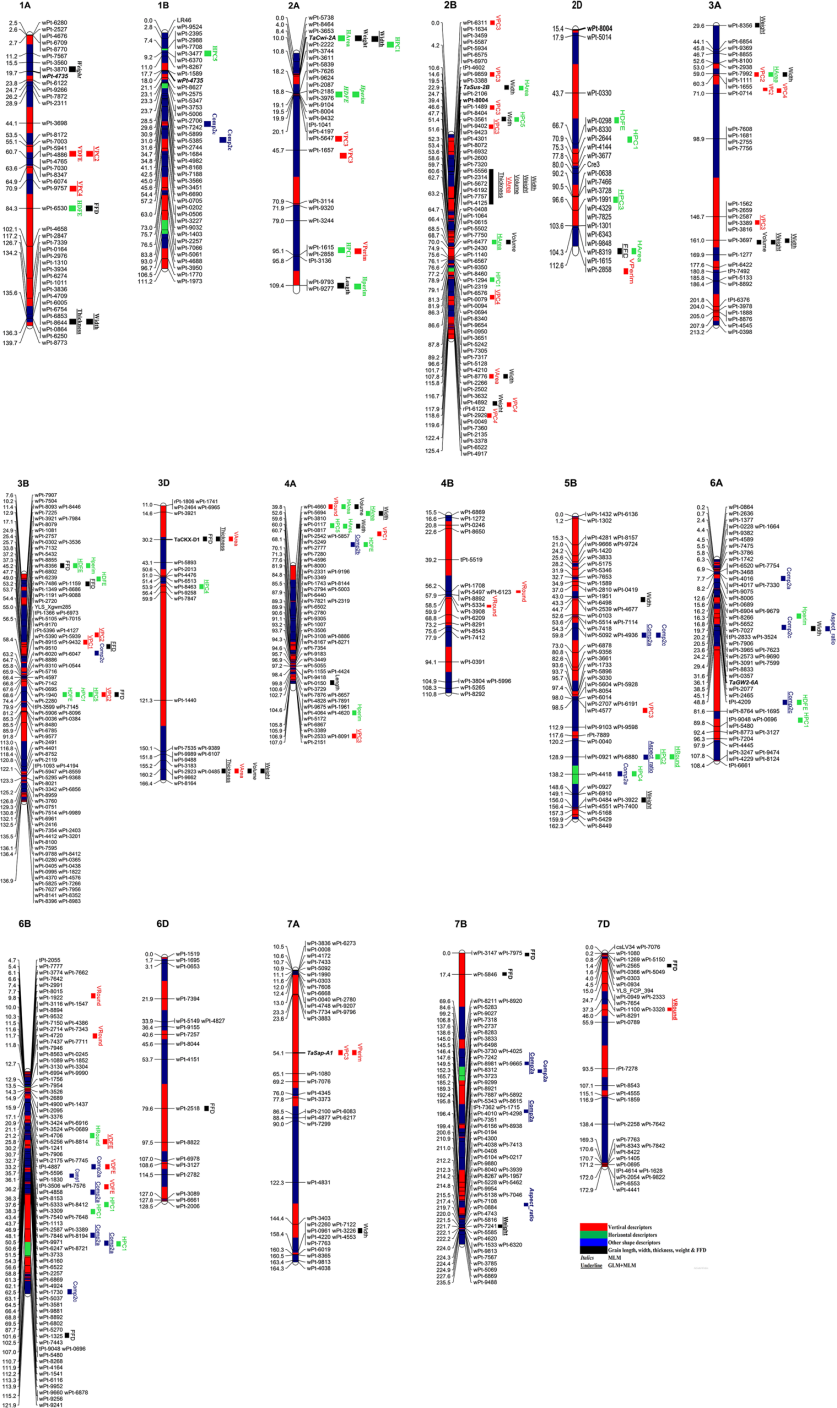
**DArT consensus linkage map (Detering et al. **[39]**) of chromosomes showing marker-traits associations for grain size and shape in synthetic hexaploids wheat.** MTAs are projected as different color solid bars for which legend is given at the end of figure. See Additional file 5: Figure S6 for high resolution images of chromosomes.

**Figure 5:**
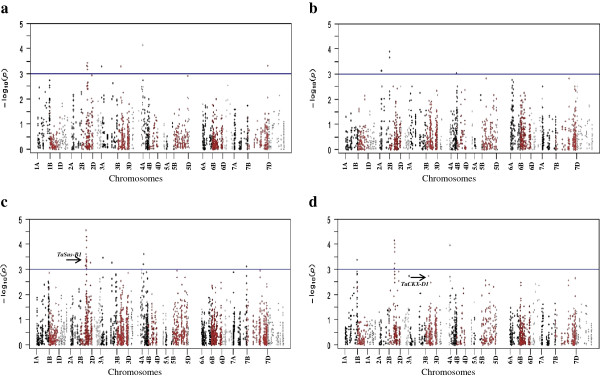
**Manhattan plots of *P *values indicating genomic regions associated with four grain morphology traits a) thousand kernel weight, b) grain length, c) grain width, and d) grain thickness. ***x*- axis shows DArT markers along each wheat chromosome; *y*- axis is the –log10 (*P*-value), horizontal lines designate 1E-03 thresholds for highly significant associations. The association of genes *TaSus-B1***(c)** and *TaCKX-D1***(d)** with grain width and thickness are shown by black arrows, respectively.

Marker-traits associations (MTAs) for grain size and shape were identified in 231 SHWs by association mapping (AM) analysis using general linear model (GLM) and mixed linear model (MLM) approaches. MTAs for eight important grain size and shape measurements namely TKW, grain length, width, thickness, volume, VArea, HArea and FFD are given in Table 3 while the MTAs for remaining 21 shapes related characteristics are given as Additional file 4: Table S4. Frequency distribution of MTA identified by GLM and MLM model over the seven wheat linkage groups and three genomes are presented in Table 4. Chromosomal linkage groups for significant MTAs are shown in Figure 4 while the Manhattan plot of all *P* values observed in this study is presented in Figure 5.

**Table 3:** Marker-trait association (MTA) for important grain size and shape characters using GLM (Q model) and MLM (Q + K model) approach in D-genome synthetic hexaploids

**Trait**	**Marker**	**Chr**^**a**^	**Pos**^**b**^	**MAF**^**c**^	**GLM**		**MLM**		**QTL/Gene**^**d**^
					***P***	***R***^***2***^	**P**	***R***^***2***^	
FFD	wPt-6530	1A	84.3	0.4	1.07E-03	5.2			
	wPt-1301	2D	104	0.38	1.12E-03	7			MQTL19 [41]^e^
	wPt-6343	2D	104	0.32	2.52E-04	8.6	1.69E-03	7.7	MQTL19 [41]
	wPt-8356	3B	45.2	0.27	9.73E-04	5.2			*QTkw.sfr-3B.1
	wPt-1159	3B	53.2	0.35	1.64E-04	5.8	1.82E-03	4.2	
	wPt-8915	3B	58.4	0.27	1.19E-03	5.1	9.37E-04	5.6	*DREB-3B*[42]
	wPt-1940	3B	68.6	0.19	4.04E-04	5			*LDD-3B *[42]
	*TaCkx-D1*	3D	30.2	0.12	0.0029	4.1			Q56 [19]
	wPt-1325	6B	102	0.36	1.20E-03	6.7			
	wPt-2518	6D	79.6	0.36	1.06E-03	5.6			*QGyld.agt-6D
	wPt-3147	7B	0	0.44	5.34E-04	4.8			*Qyld.idw-7B
	wPt-5846	7B	17.4	0.45	1.02E-03	4.3			
	wPt-2565	7D	1.37	0.25	8.44E-04	6.6			
	*Wx-D1*	7D	.		0.0156	2.7			
HArea	*TaCwi-A1*	2A	10.5	0.25	8.00E-03	3.2			
	*TaSus-B1*	2B	51.6	0.19	0.0234	7.1			*FdGogat-B *[42]
	wPt-6477	2B	70	0.37	9.24E-04	7.6	1.69E-03	4.9	*Qyld.e3-2B
	wPt-8319	2D	104	0.26	7.46E-04	8.4			MQTL19 [41,43]
	wPt-7992	3A	59	0.42	1.18E-05	13.6	9.56E-04	9.7	*Vp1-A *[42]
	wPt-4660	4A	39.8	0.39	9.78E-04	5.1			*QGyld.agt-4A
	wPt-5694	4A	52.6	0.38	3.82E-04	8.9	8.80E-04	8.1	
	wPt-0117	4A	60	0.36	3.22E-04	9			*QGwt.crc-4A
Length	wPt-9277	2A	109	0.33	1.23E-04	8.5			*QGwt.crc-2A; Q09 [19]
	wPt-9793	2A	109	0.32	2.21E-04	8.4			*QGwt.crc-2A
	wPt-0150	4A	99.8	0.12	9.01E-04	8.9			
Thickness	wPt-8644	1A	136	0.27	4.24E-04	5.8	1.24E-03	5.1	Q17 [19]
	wPt-5556	2B	60.6	0.44	6.77E-05	7.5	9.22E-04	5.4	*Qyld.crc-2B
	wPt-4125	2B	63.2	0.43	2.04E-04	6.6			
	wPt-5672	2B	63.2	0.44	1.32E-04	6.8	1.61E-03	4.9	
	wPt-6192	2B	63.2	0.36	5.88E-04	6.6			
	wPt-7757	2B	63.2	0.44	9.62E-05	7.2	1.18E-03	5.2	
	*TaCkx-D1*	3D	30.2	0.12	4.03E-05	7.6	3.03E-05	6.3	Q56 [19]
	wPt-0485	3D	160	0.39	1.06E-04	9.8	1.91E-03	7.2	*Qyld.e4-3D
	wPt-2923	3D	160	0.4	1.12E-04	9.7			*Qyld.e4-3D
VArea	wPt-5556	2B	60.6	0.44	2.30E-05	8.6	3.04E-04	6.6	
	wPt-4125	2B	63.2	0.43	9.68E-05	7.4	9.07E-04	5.6	
	wPt-5672	2B	63.2	0.44	5.14E-05	7.8	5.85E-04	6	
	wPt-6192	2B	63.2	0.36	2.82E-04	7.4	1.70E-03	5.6	
	wPt-7757	2B	63.2	0.44	3.77E-05	8.1	4.55E-04	6.2	
	wPt-8776	2B	108	0.15	4.78E-04	7.4			
	*TaCkx-D1*	3D	30.2	0.12	0.0029	4.1			Q56 [19]
	wPt-0485	3D	160	0.39	2.05E-04	9			*Qyld.e4-3D
	wPt-2923	3D	160	0.4	1.96E-04	9			*Qyld.e4-3D
Volume	wPt-5556	2B	60.6	0.44	2.14E-04	6.5	8.70E-04	5.6	
	wPt-4125	2B	63.2	0.43	3.88E-04	6	1.58E-03	5.1	
	wPt-5672	2B	63.2	0.44	3.24E-04	6	1.46E-03	5.1	
	wPt-7757	2B	63.2	0.44	2.84E-04	6.2	1.17E-03	5.3	
	wPt-6477	2B	70	0.37		0	1.86E-03	4.8	*Qyld.e3-2B
	wPt-3697	3A	161	0.07	1.05E-03	7.2			
	wPt-0485	3D	160	0.39	1.03E-03	7.4			*Qyld.e4-3D
	wPt-2923	3D	160	0.4		0	1.41E-03	5.2	*Qyld.e4-3D
	wPt-4660	4A	39.8	0.39	5.67E-04	5.7			*QGyld.agt-4A
Weight	wPt-3870	1A	19.7	0.47		0	0.0094	4.8	*Qyld.e1-1A
	*TaCwi-A1*	2A	10.5	0.25	0.023	3			
	wPt-5556	2B	60.6	0.44	4.80E-04	5.5	0.00307	4.1	
	wPt-4125	2B	63.2	0.43	6.83E-04	5.2	0.00408	3.9	
	wPt-5672	2B	63.2	0.44	3.60E-04	5.6	0.00238	4.3	
	wPt-7757	2B	63.2	0.44	5.12E-04	5.4	0.00279	4.1	
	wPt-2266	2B	116	0.05	1.10E-03	6.8			
	wPt-8356	3A	29.6	0.27	5.00E-04	6.4	0.00868	3.7	
	wPt-3697	3A	161	0.07		0	0.00762	4.9	
	wPt-0485	3D	160	0.39	6.76E-05	10.4	0.00441	6	*Qyld.e4-3D
	wPt-2923	3D	160	0.4	7.35E-05	10.3	0.00477	5.9	*Qyld.e4-3D
	wPt-8164	3D	166	0.41	7.35E-05	10.2	0.00329	6.7	*QGyld.agt-3D
	wPt-0484	5B	156	0.08	1.19E-03	6.7	0.00869	5	Q30 [19]
	wPt-7241	7B	222	0.37	4.69E-04	8.7	0.00917	5.1	
Width	wPt-8644	1A	136	0.27	4.24E-04	5.8	1.24E-03	5.1	Q17 [19]
	*TaCwi-A1*	2A	10.5	0.25	0.0146	2.7	0.0187	2.8	
	*TaSus-B1*	2B	51.4	0.19	0.003	6.6			*FdGogat-B *[42]
	wPt-3561	2B	51.4		7.25E-04	7.9			*FdGogat-B *[42]
	wPt-5556	2B	60.6	0.44	2.78E-05	8.5	2.49E-04	6.8	
	wPt-4125	2B	63.2	0.43	1.34E-04	7.1	7.70E-04	5.8	
	wPt-5672	2B	63.2	0.44	7.00E-05	7.6	5.01E-04	6.2	
	wPt-6192	2B	63.2	0.36	3.28E-04	7.3	1.63E-03	5.7	Q42 [19]
	wPt-7757	2B	63.2	0.44	4.99E-05	8	4.13E-04	6.3	
	wPt-8776	2B	108	0.15	5.30E-04	7.4			*QTkw.sfr-B1 *[44]
	wPt-7992	3A	59	0.42	3.37E-04	9.2			*Vp1-A *[42]
	wPt-3697	3A	161	0.07	5.35E-04	7.9			*Qyld.e4-3D
	wPt-0485	3D	160	0.39	1.09E-03	7.2			*Qyld.e4-3D
	wPt-2923	3D	160	0.4	9.61E-04	7.3			
	wPt-5694	4A	52.6	0.38	6.25E-04	8.3	1.45E-03	7.3	
	wPt-0117	4A	60	0.36	2.44E-04	9.4			*QGwt.crc-4A
	wPt-6498	5B	43.3	0.44	1.11E-03	5.6			*Qyld.e11-5B
	*TaGW2-A1*	6A	36.1	0.18	0.0197	7.5			Q06 [19]
	wPt-3226	7A	158	0.24	7.75E-04	5.8			*Qyld.e15-7A

^a^*Chr* Chromosome, ^b^*Pos* the marker position on the linkage map based on Detering et al. [39], ^c^*MAF* Minor allele frequency, ^d^*QTL*/*Gene* the previously reported QTLs or genes within the same chromosomal regions with reference. ^e^MQTL refers to meta-QTLs for grain yield related traits described in Zhang et al. [41]. ^*^QTLs without reference are extracted from the consensus maps of individual chromosomes as of June 2011 (http://ccg.murdoch.edu.au/cmap/ccg-live/cgi-bin/cmap/viewer?).

**Table 4:** Distribution of marker-trait associations (MTAs) identified using GLM and MLM models in D genome synthetic hexaploids

						**Homoeologous groups**^**e**^							**Genome**^**f**^		
**Trait**	**Total**	**GLM**^**a**^	**MLM**^**b**^	**Both**^**c**^	**FDR**^**d**^	**1**	**2**	**3**	**4**	**5**	**6**	**7**	**A**	**B**	**D**
HPC1	10	9	1	-	-	-	-	1	-	5	4	-	4	5	1
HPC2	1	-	-	1	1	-	-	-	-	1	-	-	-	1	-
HPC3	1	-	-	1	-	-	1	-	-	-	-	-	-	-	1
HPC4	2	1	1	-	-	-	-	1	-	1	-	-	-	1	1
HPC5	4	3	-	1	1	1	1	1	1	-	-	-	1	3	-
VPC1	2	1	-	1	1	-	-	1	1	-	-	-	1	1	-
VPC2	5	2	-	3	1	1	-	-	4	-	-		3	2	-
VPC3	15	11	-	4	2	-	9	1	2	2	-	1	6	9	-
VPC4	5	1	2	2	1	1	3	1		-	-	-	2	3	-
VPC5	-	-	-	-	-	-	-	-	-	-	-	-	-	-	-
HArea	8	5	-	3	-	-	4	1	3	-	-	-	5	2	1
HPerim	6	5	1	-	-	-	3	1	1	-	1	-	5	1	-
Length	3	3	-	-	-	-	2	-	1	-	-	-	3	-	-
Width	19	11	-	8	2	1	9	4	2	1	1	1	8	9	2
HRound	2	1	-	1	1	-	-	-		1	1	-	-	2	-
HDFE	9	6	2	1	1	1	3	3	1	-	1	-	5	3	1
Aspect ratio	3	1	1	1	-	-	-	-	-	1	1	1	1	2	-
VArea	9	4	-	5	2	-	6	3	-	-	-	-	-	6	3
VPerim.	4	4	-	-	-	-	3	-	-	-	-	1	2	-	2
Thickness	9	3	-	6	1	1	5	3	-	-	-	-	1	5	3
VRound	13	11	-	2	-	-	-	-	4	-	7	2	1	10	2
VDFE	5	4	-	1	-	1	-	-	-	-	4	-	1	4	-
Volume	9	3	2	4	-	-	5	3	1	-	-	-	2	5	2
FFD	14	11	-	3	-	1	2	5	1	-	2	3	1	7	6
Comp1	1	1	-	-	-					-	1	-	-	1	-
Comp2a	15	8	1	6	-	-	-	-	-	2	8	5	1	14	-
Comp2b	1	-	-	1	-	-	-	-	1	-	-	-	1	-	-
Comp2c	8	7	-	1	-	3	-	1	-	1	3	-	2	6	-
TKW	14	1	2	10	-	1	6	5	-	1	-	1	4	7	3
Total	197	117	13	66	14	12	62	35	23	16	34	15	60	109	28

^a^*GLM* MTAs identified by general linear model only, ^b^*MLM* MTAs identified by mixed linear model only, ^c^*Both* MTAs identified by both model, ^d^*FDR* Number of MTAs passed false discovery rate test, ^e^Number of MTAs present in each of the seven wheat homoeologous group, ^f^Number of MTAs present in each of the three wheat genomes.

The GLM approach identified 197 DArT loci on 60 chromosomal regions to be associated with grain phenotype traits; this was reduced by 60% (79 loci over 31 chromosomal regions) when analyzed using MLM model (Tables 3 and S4). Using GLM, MTAs for grain size and shape were identified on all chromosomes except for chromosomes 1D, 4D and 5A. Maximum number of MTAs (21) were found on chromosome 2B followed by 3B (15), while only one MTA was found on chromosome 6D. Maximum numbers of MTAs (109) were identified on the B genome followed by A genome (60), with the D genome exhibiting the least MTAs (28).

In total, 79 DArT markers on 31 chromosomal regions were associated with 23 grain size and shape traits using MLM approach. Among the significant MTAs, 43 markers represent direct measurements including TKW, grain area, thickness, width and FFD. Out of 79 significant MTAs, only 14 passed the FDR test out of which only three markers (wPt-5556, wPt-5672, wPt-7757) represented direct grain size measurement i.e. grain width and vertical area. These markers are on same chromosomal region (2B, 60–63 cM) and are in significant LD (*r*^*2*^ = 0.45). Phenotypic variability explained by most of the markers were greater than 5%. The marker wPt-8915 on chromosome 3B possessed the maximum phenotypic variation (13.6%) for VPC1.

MTA analysis also revealed that 35 DArT loci were associated with multiple traits. Multiple trait associations ranged from two to five traits per DArT locus. Twenty one, six, one, and seven DArT loci were associated with two, three, four, and five traits, respectively.

Association of markers for known genes controlling grain size and weight like *TaCwi-2A*, *TaSus-6B, TaCKX-6D* and *TaGW2*-*2B* were also validated in this study as indicated in Table 3. The results confirmed the validity of AM approach for alleles of these genes in SHWs. Alleles for the *TaCwi-2A* gene were significantly associated with TKW, grain width and horizontal area with *r*^*2*^ of 3.2% and 3.0%, respectively. Similarly, allelic variations for *TaSsus-B1* were found to be associated with grain width and horizontal area with *r*^*2*^ of 6.6% and 7.1% respectively. Allelic variation for gene encoding cytokinin oxidase/reductase, *TaCKX-D1*, found to be associated with grain thickness, vertical area of grain and vertical deviation from ellipse (VDFE). This strong association for all traits including vertical dimensions revealed the effect of the gene on variability for this dimension of the grain. Similarly, aspect ratio was found to be strongly associated with *TaGW2-6A* gene.

### Relationship between grain phenotype and number of favored alleles

**Figure 6:**
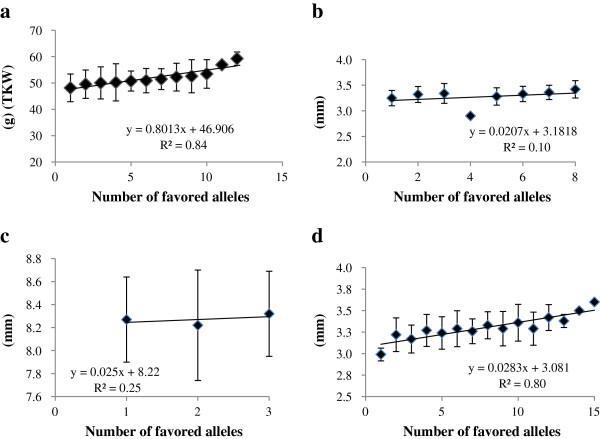
Linear regressions between number of favored alleles and mean phenotypic effect on a) Thousand kernel weight b) Grain thickness c) Grain length d) Grain width.

A linear relationship was observed for grain length, width, thickness and weight, where the addition of every favorable allele in a variety additively contributed to enhance the phenotype (Figure 6). However, there was only one SHW having four favorable alleles for grain thickness which reduced the linear correlation and resulted in negative interaction. Similar trend was observed for grain length, where only 3.2% of the SHWs have two favorable alleles. The number of markers associated with TKW and grain width was relatively high as compared to grain length and thickness.

### Functional analysis of DArT clones associated with grain phenotype

Sequences of 107 DArTs were used as a query for similarity search using BLASTX algorithm. In many cases, sequences were very short therefore matches were also searched in International Wheat Genome Survey Sequence (IWGSS) database. If a longer genomic clone was identified, it was used as a query in Blast2Go software. Blast search gave positive result for 73 DArT clones, which therefore represent putative expressed sequences. However, putative biological function could be predicted for 20 DArT loci (Additional file 6: Table S5). The remaining putatively expressed sequences corresponded to EST or protein sequences without functional annotation or known domains. Seven of such DArTs (wPt2533, wPt-8091, wPt-3389, wPt-9423, wPt-9402, wPt-1489, wPt-8087) had association with a grain shape parameter (VPC3) which has significant negative effect on grain weight.

For D-genome specific DArTs, 12 out of 16 sequences were traced for their corresponding scaffold and putative function of 8 DArT clones could be determined (Table 5). Four DArT sequences were found in proximity of expressed regions, however protein function was uncharacterized. Putative function of four DArT sequences on chromosome 3D associated with relatively important grain phenotypes are very important as the members of receptor like kinase (LrK) family. These results are important because these regions may carry novel alleles for grain phenotype and SHWs can facilitate their identification and subsequent introgression to bread wheat to enhance grain yield.

**Table 5:** **Co-localization of traits associated DArT markers on D-genome with *Ae. tauschii *draft genome sequence data (Jia et al. 2013) **[45]

**DArT**	**Trait associated**	**Chr**	**Pos**	***Ae. tauschii *draft genome sequence accessed 2013/10/26**		
				**Scaffold**	**E-value**	**GO**
wPt-2644	HPC1	2D	70.93	46122	0	P-loop containing nucleoside triphosphate hydrolase
wPt-1301	FFD	2D	103.56	12384	7E-214	Topoisomerase DNA binding C4 zinc finger
wPt-6343	FFD	2D	103.56	12384		Topoisomerase DNA binding C4 zinc finger
wPt-8319	HArea	2D	104.27	59412	3E-12	UCP
wPt-1615	VPerim	2D	112.64	15328	3E-94	UCP
wPt-2858	VPerim	2D	112.64	115328	8E-42	UCP
wPt-8463	HPC4	3D	53.86	60730	2E-12	Transmembrane helicase
wPt-0485	Thickness	3D	160.16	73711	5E-180	Putative cysteine-rich receptor-like protein kinase 39
wPt-2923	Weight, Thickness, Vol, Varea, Width	3D	160.16	73711	5E-180	Putative cysteine-rich receptor-like protein kinase 39
wPt-8164	Weight, Thickness, Vol, Varea, Width	3D	166.43	73711	2E-134	Wall-associated receptor kinase 5
wPt-2565	FFD	7D	1.37	126470	0	Disease resistance protein RPP13
wPt-1100	Vround	7D	37.25	19484	6E-174	UCP

*GO*: Gene ontology; *UCP*: Uncharacterized protein.

## Discussions

### Phenotypic evaluation of grain morphology using digital imaging

Seed shape and size are among the most important agronomic traits due to their significant effect on grain weight, milling yield, and market price. Manual measurement methods have limits to the number of data, the quality of measurements, and the variety of shape data that can be gleaned. By contrast, computational methods using DI technology could enable us to automatically measure robust size descriptors (grain length, width, perimeter and area) and Elliptic Fourier descriptors (EFDs) capturing shape variation such as roughness, asymmetric skewing or other two dimensional aspects not encompassed by axes or distinctions in overall object area [15]. Only few studies are available based on DI analysis of seed size and shape in wheat [15,16,19,26,29]. Among these studies, Gegas et al. [16], Williams et al. [15] and Williams and Sorrells [19] used shape variations as targeted traits influencing grain size and weight and results are comparable to our work. In this study, low correlations between the major grain dimensions and EFDs indicate that different aspects of grain morphology were captured by each phenotyping method and likely could be selected independently. Because EFDs were more highly correlated with TKW and grain length than other traits, therefore, it would be preferred if kernel shape were used in selection to increase length and TKW. The correlations between EFDs and TKW suggest that they are able to relate the uniformity and smoothness of the kernel to grain weight because roughness or shriveling would be expected to reduce the ratio of internal volume to surface area of the kernel. Use of EFDs recorded from kernels imaged on end (vertical images in this study) also can characterize variation in the depth or angle of a wheat seed’s crease which will impact the volume to surface area relationship of a grain.

A large number of significant correlations were observed for remaining size and shape traits (Table 1). For the ease of understanding we only discussed important relationships that give us new insight into the complex composition of grain size and shape components. In the nutshell: i) grain roundness has significant negative relationship with grain length indicating both traits influencing grain weight independently; ii) horizontal and vertical deviations from optimal ellipse were positively correlated with grain length and width, respectively, indicating deviation from the ellipse enhances grain length and width, and ultimately TKW; iii) grain length and width had slightly significant positive correlation indicating the possibility of finding some SHWs having wider and lengthy grains simultaneously which may lead to above the average TKW. This possibility of finding co-localized QTLs influencing grain length and breadth is expected and discussed below. However, grain width had more positive impact on TKW as compared to grain length. Although previous studies reported moderate correlations between grain weight, length, and width with *r* = 0.51–0.68 [30], and *r* = 0.21–0.75 [27], our results were in agreement with Lee et al. [46], who reported strong correlation (*r* = 0.83) between kernel weight and size. Studies have shown that kernel weight was positively correlated with grain yield [47] and kernel growth rate [48]; however, Xiao et al. [29] found TKW less correlated with grain yield in 1B.1R × non 1B.1R crosses across environments.

All previous reports described grain size and shapes emerged as independent traits in primitive and improved wheat germplasm [16], similar to the results obtained in this study using the D genome synthetic hexaploids. However, the significant reduction of phenotypic variation in grain shapes in breeding germplasm pool is probably as a result of relatively recent evolutionary and domestication bottleneck. As a consequence, the phenotypic variability offered by SHWs may fill the gap and is a good choice germplasm which can be used to improve grain weight of wheat, hence enhancing grain yield.

The association of grain size and shape descriptors with TKW was further resolved by path coefficient analysis which depicted the phenotypic model with more precision. This revealed that grain thickness has maximum direct effect on grain weight followed by VArea, whereas HArea has relatively less direct effect on grain weight. Some principal components like HPC1, HPC2, VPC3, and VPC4 have direct negative effect on grain weight and loci harboring their control should undergo negative selection in order to get superior grain weight genotypes. The efficiency of indirect selection depends on the correlation between a selected trait and a target trait as well as the heritability of the selected trait. Gegas et al. [16] confirmed that kernel size and shape were largely independent traits in a study of six wheat populations. The results showed that the phenotypic correlations among these traits were caused by closely linked genes or genes with pleiotropic effects.

### Genetic diversity and population structure in synthetic hexaploid wheats

Genetic diversity within *Ae. tauschii* and synthetic hexaploids have been studied using several marker systems [4]. Recently, Sohail et al. [10] analyzed the diversity using 4,449 polymorphic DArT markers and found the diversity of *Ae. tauschii* ssp. *strangulata,* the origin of D genome of bread wheat, contains only a limited part of whole diversity of *Ae. tauschii.* Thus, SHWs produced by crossing between tetraploid wheat and any subspecies of *Ae. tauschii* include untapped amount of genetic variation in which useful genes for bread wheat breeding must be present. Our results indicate that five substructures were appropriate in delineating the population structure within the SHWs used in this study. The assignment of the SHWs to the five subgroups was largely in agreement with their *Ae. tauschii* parent and less so with the durum parent. Recently, Mulki et al. [9] studied a wide array of synthetic hexaploids and indicated the presence of seven substructures were appropriate in delineating the population structure. The minor difference in the results may be attributed to the higher number of accessions used as compared to this study. The frequency of *Ae. tauschii* accessions amongst the SHWs varied from one to a maximum of five while the durum elite lines ranged from 1 to 45, an indication of the complexity of the crosses. It has been suggested that the STRUCTURE algorithm does not converge to an optimal K when complex genetic structures exist, such as strong relatedness within some germplasm [49].

### Linkage disequilibrium patterns

Linkage disequilibrium is influenced by recombination rate, allele frequency, population structure and selection [50]. In this study, the LD generally decreased with the increase of genetic distance with very strong LD between pairs of loci observed at genetic distances of up to 9 cM, suggestive of LD maintained by genetic linkage. Our results are consistent with those reported by previous studies in wheat. In a similar study using a subset of 91 SHWs, Emebiri et al. [51] reported that the general trend was high LD up to 15 cM, and a decline thereafter. LD was estimated to extend to about 10 cM among 43 United States bread wheat elite varieties and breeding lines [52]. Crossa et al. [53] reported that some LD blocks extended up to 87 cM in a set of 170 bread wheat breeding lines. Breseghello and Sorrells [40] suggested that LD may differ among populations and may need to be evaluated for each population on a case-by-case basis. Nevertheless, it is important to characterize germplasm for examining the extent of LD to study the genetic diversity. Overall the observed LD was low in SHWs and only ~10.2% of the marker pairs reached the threshold of 0.2 *r*^*2*^ value in the collection (N = 13453; marker pairs). Generally self-fertilization leads to a more extensive LD due to the several reduced effective recombination levels [50]. The lower values of LD observed in SHWs are in concordance with what has been previously reported by Chao et al. [54] using the SNP marker system. They reported that CIMMYT wheat populations with the lowest LD among completely linked loci and the slowest rate of LD decay was possibly a consequence of an intensive use of synthetic wheat lines. Synthetics wheats and their derivatives have greatly increased genetic diversity in hexaploid wheat, particularly in the D-genome [55]. A similar case is observed in these SHWs where unusual patterns of LD, rate of LD decay and lower pairwise *r*^*2*^ values are attributed to the genomic constitution of the germplasm. It is well known that the introduction of new haplotypes from divergent population can increase the extent of LD [56].

### Marker-trait associations and co-linearity with identified QTLs

The MTAs identified in this study, can be categorized as those affecting (1) individual dimensions of the seed and TKW, (2) multiple dimensions of the seed (meaning a single QTL that affects more than one dimension of the seed, such as length and width simultaneously), and (3) individual dimensions of the seed but not TKW. This study is the first report in using association mapping for grain size and shape that employed quantitative photometric measurements. In total, 38 MTAs for grain length, width, thickness, and TKW are relatively most important due to their immediate effect on enhancing grain yield. The co-linearity of MTAs of different traits was observed on chromosomes 1A, 2B, 3A, 3D, and 5B and indicated these regions as stable. A complete region on chromosome 2B from 51 to 69.9 cM harbor 31 MTAs of important grain phenotypes which is strong evidence of the presence of some functional genes within this proximity affecting grain phenotype. Previously, 3 meta-QTLs were identified on chromosome 2B [41], but none can be co-localized within this region. The proximity of *TaSus-*B1 on chromosome 2B is within some of the MTAs identified in this study (Table 4). The co-linearity of some of the important genes and QTLs revealed the presence of *Ppd-B1*, *Q.Yld.crc-2B* and *QTkw.sfr-2B* within proximity of this region. The selection based on these DArT markers can result in selection of SHWs carrying better grain size and shape phenotypes which can be exploited for wheat genetic improvement.

Previously, only two association mapping studies are available solely focusing on mapping of grain weight QTLs [22,31]. None of two studies used DArT markers, hence, it will be difficult to align and compare QTLs detected by these studies. However, we were able to identify five loci within proximity of QTLs identified by Williams and Sorrells [19] using consensus DArT map information [39]. These QTLs include Q56 (FFD, grain thickness, grain area) on chromosome 3D, Q09 (grain length) on chromosome 2A, Q17 (grain thickness, width) on chromosome 1A, Q30 (TKW) on chromosome 5B and Q42 (grain width) on chromosome 2B. Previously, QTLs affecting seed size have been identified across all chromosomes of wheat, with varying degrees of effect seen for individual QTL [16,26-28,30], and many of them were found within same regions identified in the present study (Table 3).

It is expected that 26 MTAs present on chromosomes 2D, 3D, 5D, 6D, and 7D may have novel allelic variability for measured traits. Horizontal area and FFD were found to be associated with markers present on same genetic region (104 cM) on chromosome 2D indicating the relative importance of this region underlying grain size and weight. Zhang et al. [41] identified a meta-QTL related to grain weight within the same region. Chromosome 3D appeared to have two genomic regions associated with TKW, grain thickness, width, volume and VArea. Additionally, the known wheat grain weight encoding gene, *TaCKX-D1*, was found to be associated with VArea, grain volume and VDFE indicating the contribution of this locus to grain weight may be through the route to enhance vertical area of grain. Similarly, five very important traits (FFD, grain width, thickness, TKW and VArea) were found to be clustered on the distal portion of chromosome 3D. Several QTLs related to grain weight have been identified on chromosome 3D and available in literatures [42].

Haplotype analysis of other known grain weight encoding genes, *TaCwi-2A, TaSus-6B, TaGW-2B* and *TaSAP-A1*, also dissected their potential role to enhance grain weight through different photometric measurements of grain size and shape. MTAs solely for TKW, grain length and width were identified on chromosomes 1A, 2B, 3A, 3B, 3D, 4A, 5B, 7A and 7B. Several QTLs were previously reported for kernel width and length on different chromosomes; for example, Campbell et al. [57] reported QTLs on chromosomes 1A, 2A, 2B, 2DL, and 3DL. Breseghello and Sorrells [26] reported QTLs on 1B, 2D, and 5B. Sun et al. [27] reported QTLs on chromosomes 4A and 6A which were absent in this study. Xiao et al. [29] identified a cluster of QTLs for grain length, width and weight on chromosome 6D, which also remained absent in this study. The justified reason for the absence of these QTLs is the very different genetic background of SHWs having A and B genomes from durum wheats and D genome from wild accessions of *Ae. tauschii*. Therefore, the identification of several QTLs is suggestive to be the novel addition to existing information and in case of co-linearity with existing QTLs, SHWs may carry new alleles.

Quantitative analyses of the photometric data revealed that grain size and shape are largely independent traits. This is unlikely to be the result of artificial selection during breeding since size and shape are also independent variables in primitive wheat. At the developmental level, this phenomenon may reflect differential modulation in growth (or growth arrest) along the main axes of the grain at different developmental stages. The notion that certain developmental constraints during grain growth could lead to morphological changes is further corroborated by recent studies on grain size/shape genes in rice [58,59]. The *GS3* locus was found to have major effects on grain length and weight and smaller effects on grain width [60], and the longer grains can be attributed to relaxed constraints during grain elongation [59]. The *GW2* gene was shown to alter grain width and weight and to lesser extend grain length owing to changes in the width of the spikelet hull [58]. Similarly, the *SW5* gene has been reported to affect grain width by modulating the size of the outer glume [61].

The results of our study demonstrated the value of genome-wide association mapping for identifying MTAs for grain size, shape and weight using genetic resources such as the SHWs. Given the diversity of MTAs identified, the SHWs possessing potentially novel alleles at different genomic regions could be used as parents in a marker-assisted backcrossing scheme to develop genotypes with higher grain weight, hence high yielding, in elite wheat backgrounds. For potentially new loci associated with grain phenotype, the development of appropriate genetic stocks using bi-parental populations, backcross families, near-isogenic lines and physical and chemical mutagenesis would enable appropriate delineation of the importance of these loci in enhancing grain weight. The DArT marker clones are almost sequenced and information is available in public domain that can assist geneticists to convert DArT into STS markers which would facilitate the incorporation of the favorable loci into elite wheat germplasm.

### Relationship between number of favorable alleles and grain phenotypes

One of the relative advantages of AM is the validation of favored alleles in natural germplasm collection [22,40]. Zhang et al. [62] found that allele *Xgwm130*_*132*_ underwent very strong positive selection during modern breeding. *Xgwm130* maps between *Xgwm295* and *Xgwm1002*, with a genetic distance of 1.1 cM from *Xgwm295*. Similar results were obtained for *TaSus-B1* gene for TKW, where most of the Chinese wheat germplasm carried favorable allele indicating the high selection pressure [33]. Thus, the identification of favored alleles will help in choosing parents for crossing programs, to ensure maximum levels of favored alleles across sets of loci targeted for selection, and to promote fixation at these loci [63]. Whereas linear correlations between major grain phenotypes (TKW, grain length, width and thickness) and favored alleles indicate the additive effects of QTLs or genes, the possibility of other genetic effects should not be discounted. However, powers to detect allelic effect reduce when numbers of germplasm lines are very few (Figure 6b,c).

One interesting phenomenon in wheat is that genes or markers associated with yield vary across latitudes, such as *TaSus2* on chromosome 2B [33], *TaGW2* on chromosome 6A [34] and gpw7596 (EST-SSR) on chromosome 7B [64]. Favored alleles usually occur at relatively lower latitudes. This might indicate that the functional genes at these loci, including mapped alleles and those linked with markers, might be responsive to sunlight and temperature during the growing season [58,65]. Recently, Jones et al. [66] devised a strategy to exploit *Ae. tauschii* diversity for wheat improvement in relation to climatic and environmental conditions of a specific geography. This informed and rational strategy can be applied to SHWs by identifying the *Ae. tauschii* accessions in the pedigree of SHWs lines with desirable characteristics. This will enhance the breeding values of SHWs and breeders will be able to offer novel diversity tailored to the environment in any regional breeding program. Nevertheless, current results are encouraging and wider options are available to exploit SHWs to enhance grain yield.

### Functional analysis of trait associated DArTs and draft genome sequence of *Ae. tauschii*

DArT markers have been widely used for different studies in many plant species including wheat. For many years they have been used as anonymous markers, however, the acquisition of sequence knowledge of DArT markers made them useful tool for many studies such as co-linearity studies, fine mapping of loci of interest, and identification of candidate genes in association mapping. The *in silico* identification of putative function of DArT loci associated with grain phenotype is a step forward towards exploitation of these loci for practical wheat improvement. Nevertheless, many of the DArT sequences blasted for the similarity search did not show positive results or in some cases identified genes of unknown function. However, in some cases results are encouraging. The medium to low positive results through blast analysis in this study are in agreement with Tinker et al. [67] where only 40% of the DArT sequences showed significant blast similarities to the genes in public databases. However, results were slightly higher for wheat DArT sequences and 64% of them matched with the genes in public databases [68]. In the present study, about 75% of the sequences displayed significant blast similarities and 32% of the sequences were fully annotated. The cluster of sequences of DArT markers on chromosome 2B translated into genes with valid biological functions and may be important candidates for future studies. Similarly, some grain shape parameters (like VPC3) have negative effect on grain trait and the down regulation of predicted biological function of such DArT sequences (wPt-2533, wPt-3389 etc.) may be the proper interpretation of the results. Overall, the knowledge of the functional meaning of these widespread markers will provide a very useful tool for the identification of candidate genes for traits under investigation.

The strategy here for the functional analysis of D-genome specific DArTs was slightly different which ultimately yielded more powerful results. DArT sequences were used as query to BLAST in draft genome sequence of *Ae. taushii *[45] to locate the scaffold carrying those sequences and to identify the genes within those scaffolds. This also identified the position of scaffold on chromosome based on the genetic map provided in the supplementary information of Jia et al. [45]. The candidate regions within scaffolds were explored for the flanking genes and almost all queries resulted in positive results. A summary of the results and the genes present in flanking sequences are depicted in Table 5. The strong association of markers wPt-8463, wPt-0485, wPt-2923, and wPt-8164 with several grain phenotype parameters and presence of some important genes with valid biological functions make them priority candidates for the fine mapping and subsequent cloning of the genes responsible to enhance grain size and weight. Similar is in the case of other D-genome specific DArT sequences. Overall, this approach proved to be very useful for targeting sequences that might be orthologous to genes in other cereals. Marone et al. [68] used similar approach to identify the genomic regions having NBS-LRR domain superfamily encoding tolerance to biotic stresses in plants, while more than 61 DArT sequences showed significant similarity to the gene sequences in the public databases of model species such as *Brachypodium* and rice [69]. Similarly, the DArT markers associated with insect pest resistance were also searched in different bioinformatics databases to assign the translating function to the sequences found similar [70]. Webster et al. [71] used the specific WECPDF domain within cell wall invertase gene (*IVR1*) as query to search for its homologues in wheat genome survey sequence database and found five potential isoforms on multiple chromosomes. Conclusively, this approach proved to be very useful and may serve as template for gene cloning and further deployment in wheat breeding.

## Conclusions

The integrated uses of phenomics, genomics and bioinformatics have facilitated the identification of several genomic regions and their putative functions to enhance grain size and weight in SHWs. The major loci revealed in this study may be of practical value for further improving wheat grain size as a conduit to enhance productivity. Exploiting the unique genetic diversity of the synthetics has a greater comparative advantage over conventional diversity as the alien D genome accessional novel input so far is minimal in wheat varieties.

## Methods

### Plant material

Synthetic hexaploid wheats were developed at International Maize and Wheat Improvement Center (CIMMYT) by artificially crossing the elite tetraploid wheat cultivars or their advanced breeding lines (*Triticum turgidum,* 2n = 2× = 28, AABB) with different accessions of *Aegilops tauschii* (2n = 4× = 14, DD). The F_1_ hybrids (2n = 3× = 21, ABD) produced as a result of these crosses, were treated with colchicine which caused chromosome doubling and formed fertile hexaploid wheats. In this study, 231 D-genome synthetic hexaploids developed from the combinations of 44 durum wheat varieties and 196 *Ae. tauschii* accessions (Additional file 7: Table S1) were used.

### Phenotyping

Digital imaging (DI) based phenotyping of grain size and different dimensions were employed for all the SHW genotypes grown in field conditions for two years, i.e. 2010–2011 and 2011–2012. These genotypes were planted in National Agriculture Research Center (NARC) Islamabad, Pakistan (33°43′N 73°04′E). Each genotype was planted in two 2-m rows spaced 30 cm apart. The field management followed the local normal agricultural practices. All the genotypes were photographed using digital camera. Twenty five sound and well developed seeds of each genotype were visually selected. Seeds were placed horizontally and vertically with equal distances on black paper to provide color contrast (Additional file 8: Figure S2). Two photographs were taken of quality ~40 pixels/mm. All the photographs were named according to genotype accession number and planting year.

### Image editing

After renaming images, all images were cropped to include only kernels and size standard using IrfanView software (http://www.irfanview.com). Images contrast and brightness was also enhanced to reduce the edge detection errors from shadowing. All the editing was performed using ‘Batch conversion’ command of software.

### ImageJ analysis

ImageJ software developed by National Institute of Health (NIH), USA, performs object counts and two-dimensional measurements of each object directly from JPEG files. Image files were opened in ImageJ as an image stack and size standard was selected to set scale. Images were adjusted to color threshold to avoid measurement of any false positives. To derive quantitative measures from adjusted images, a global scale was set using the size standard included with each photograph so that ImageJ could calculate actual distance based on pixel measurements. The ‘Count Object’ command was used to return values for four primary measures including major axis, minor axis, area, and perimeter of each grain (Additional file 9: Figure S3). For H images, the major axis corresponded to grain length and minor axis corresponded to grain width. For V images, same process was repeated with the major axis corresponding to grain width and minor axis corresponding to grain thickness. ImageJ output for the measures of image sets were exported to a spreadsheet where values for seed images with poor outlines were removed based on visual observation.

### Other shape derivatives

Several other derivatives of shape were measured using the formulas mentioned in Additional file 10: Table S3. These derivatives include factor from density (FFD), volume of seeds (VOL), aspect ratio (ASPECT), horizontal and vertical deviation from optimal ellipses (HDFE and VDFE).

### Elliptic Fourier descriptors

Apart from the different dimensions mentioned above, the different aspects of shape are also described by Elliptic Fourier descriptors (EFD) that are not described by conventional photometric measurement [15]. These descriptors also provide robust quantitative measures of plant organ shape. EFDs are generated by superimposing the outline of a shape onto a coordinate plane then converting the outline into a numeric description that can be subjected to principle component analysis (PCA). Individual PCA scores can then be used directly as phenotypic data for genetic analyses (Additional file 11: Figure S4). All measurements were performed by SHPAE package which is combination of several applications.

### Genotyping for DArT markers

Genomic DNA of all SHWs was extracted and was sent to Triticarte Pty. Ltd. Australia (http://www.triticarte.com.au) for genotyping, as a commercial service provider for DArT markers. DArT is an array-based genotyping technology which generates DNA markers that are binary and dominant. The basis of polymorphisms is single nucleotide polymorphisms (SNPs) and insertion/deletions (InDels) at restriction enzyme cutting sites and large InDels within restriction fragments [72]. A high-density DArT array was used and 1200 DArT markers were scored.

### Allelic and haplotype effects of some valid seed weight contributing genes

In addition to DArT markers, some specific markers influencing grain weight in wheat were also applied to assess their allelic and haplotype effects. Two functional markers CWI21 and CWI22 were developed to validate alleles *Tacwi-A1a* and *Tacwi-A1b* associated with low and high TKW, respectively [32]. Jiang et al. [33] reported that two haplotypes Hap-H and Hap-L at *Tasus2-2B* locus have significant effect on TKW in wheat. Two functional markers were developed based on the SNP present in the coding sequence of gene. These two markers were applied on all SHW to identify the relevant allele. Su et al. [34] identified a haplotype Hap-6A-A at *TaGW2-6A* locus significantly associated with wider grains and high TKW in wheat. A CAPS marker was developed generating *Taq*I recognition fragments of 167 and 218 bp in cultivars with Hap-6A-A and Hap-6A-G, respectively. Zhang et al. [35] analyzed the haplotype diversity and expression of CKX enzyme in wheat and its relationship in enhancing grain weight. They identified two haplotypes *a* and *b* significantly associated with grain weight and designed a functional marker based on 18 bp InDel in their sequences.

### Statistical analysis

#### ***Gene diversity, marker allele frequency and construction of genetic map***

Gene diversity, polymorphic information content (PIC) and marker allele frequency were calculated using PowerMarker v3.25 [73]. DArT markers with minor allele frequency of less than 5% were culled from the data set to reduce false positives. The remaining DArT markers were integrated into a linkage map by inferring marker order and position from a consensus genetic map of wheat [39] (ordering 5,000 wheat DArT markers).

### Population structure

Forty-two unlinked markers specific to all chromosomes of A, B and D genomes in all synthetic hexaploids were selected to calculate population structure. The genetic distance between two chosen markers on the same chromosome was at least 50 cM to avoid physical linkage. Population structure was estimated using STRUCTURE 2.3.3-a model based (Bayesian) cluster software [74]. The number of subpopulations (K) was set from 2–20 based on admixture and correlated allele frequencies models. For each K, 10 runs were performed separately. Each run was carried out with 30,000 iterations and 30,000 burn-in periods. A value of K was selected where the graph of *InPr* (X/K) peaked in the range of 2–20 subpopulations. For selected K again 10 runs were performed each with 100,000 iterations and 100,000 burn-in periods. An ad-hoc quantity statistic (ΔK) based on the rate of change in the log probability of data between successive K values [75] was used to predict the real number of subpopulations.

### Linkage disequilibrium

Pairwise linkage disequilibrium pattern was measured using TASSEL 2.0.1 software [76]. The comparison wise significance was computed using 1,000 permutations as implemented in TASSEL software. The position of DArT markers in terms of genetic distances (cM) were based on the consensus DArT map [39]. LD levels and the rate of LD decay were computed by calculating *r*^*2*^ for pairs of DArTs and plotting them against genetic distance. The statistical significance of individual *r*^*2*^ estimates was calculated by the exact test following Weir et al. [77]. Chromosome specific *r*^*2*^ values were plotted using the R package LDheatmap.

### Association analysis

Association analysis was performed using the general linear model (GLM) and the mixed linear model (MLM) functions of TASSEL. In GLM, a single factor analysis of variance (SFA) that did not consider population structure was first carried out using each marker as the independent variable and comparing the mean performance of each allelic class. GLM was further performed with population structure (Q matrix) integrated as covariate to correct for the effects of population substructure. Finally, the MLM accounting for both Q and family structure matrix (Kinship, K matrix) to control both Type I and Type II errors [78] was performed. To correct for multiple testing, a false discovery rate (FDR) method described [79] was used to declare significant marker-trait associations with relevant grain phenotype descriptor. The Manhattan plot was drawn using ggplot2 code in R written by Stephen Turner (http://www.statmethods.net/advgraphs/ggplot2.html).

### *In silico* functional analysis of DArT loci associated with grain phenotype

The complete sequences of DArT clones associated with traits were obtained from Triticarte Pty. Ltd. Putative functions of these loci were identified using *in silico* approach. Sequences were imported to Blast2Go software as fasta format [80] which were blasted, mapped and annotated using the standard parameters embedded in software. Annotations of the resulting proteins were confirmed or implemented by searching known domains in the Pfam database (http://www.sanger.ac.uk/resources/databases/pfam.html). Recently, the draft genome sequence of *Ae. tauschii* is reported [45], therefore, DArT clones from the D-genome were blasted in this database to narrow down their location in scaffolds and co-localization with the genes/transcription factors already annotated.

## Abbreviations

DArT: Diversity array technology; DI: Digital imaging; EFD: Elliptic Fourier descriptors; FFD: Factor from density; GLM: General linear model; GWAS: Genome wide association studies; HArea: Horizontal area; HDFE: Horizontal deviation from ellipse; HPC1-5: Horizontal principal component 1–5; HPerim: Horizontal perimeter; LD: Linkage disequilibrium; MTA: Marker-trait association; MLM: Mixed linear model; QTLs: Quantitative trait loci; SHWs: Synthetic hexaploid wheats; VDFE: Vertical deviation from ellipse; VPC1-5: Vertical principal component 1–5; VPerim: Vertical perimeter.

## Competing interests

The authors declare that they have no competing interests.

## Authors’ contributions

AR carried out the research work and drafted the manuscript. TM and AM participated in the design of the study and reviewed the manuscript. TM contributed in the Bioinformatics analysis of the sequences of DArT markers. FO and AR conducted the statistical analysis of data and FO provided intellectual support during manuscript writing. ZZ, ZH and XC conceived the study, participated in the design of experiment and revised the manuscript. All the authors read and approved the final manuscript.

## Supplementary Material

Additional file 1: Table S2 Basic statistics of grain phenotype descriptors in D-genome SHWs. Click here for file

Additional file 2: Figure S1 Frequency distribution of all traits related to grain size and shape in SHWs. Click here for file

Additional file 3: Figure S5 LD heatmap of all wheat chromosomes showing extent of pair wise linkage dis-equilibrium between DArT markers. Click here for file

Additional file 4: Table S4 MTAs identified for grain shape in SHWs. Click here for file

Additional file 5: Figure S6 DArT consensus linkage map (Detering et al. [39]) of chromosomes showing marker-traits associations for grain size and shape in synthetic hexaploids wheat. MTAs are projected as different color solid bars for which legend is given at the end of figure. Click here for file

Additional file 6: Table S5 Functional analysis of DArT associated with grain phenotype. Click here for file

Additional file 7: Table S1 Pedigree information of the SHWs used in this study. Click here for file

Additional file 8: Figure S2 Upper row: Horizontal images of synthetic hexaploid accession AUS33412, A) Original image file, B) image after color threshold to measure individual grains C) outlines created by ImageJ after measuring horizontal shape descriptors. Lower row: Vertical images of synthetic hexaploid accession AUS33412, D) Original image file, E) image after color threshold to measure individual grains F) outlines created by ImageJ after measuring horizontal shape descriptors. Click here for file

Additional file 9: Figure S3 Dimension axis and their measurement demonstrated by ImageJ software for original grain image and its fitted ellipse. Click here for file

Additional file 10: Table S3 Photometric measurements to phenotype seed design for association genetic analysis. Click here for file

Additional file 11: Figure S4 Transformation of grain shape into five principal components to generate high-throughput quantitative data suitable for genetic analysis (Accession: AUS34404). Click here for file
