# Supplementary material for: Genome-wide association for grain morphology in synthetic hexaploid wheats using digital imaging analysis
Source: BMC Plant Biol. 2014 May 9;14:128. doi: 10.1186/1471-2229-14-128 (PMC4057600; doi:10.1186/1471-2229-14-128)
Supplement: Additional file 1: Table S2 — Basic statistics of grain phenotype descriptors in D-genome SHWs. [file 1471-2229-14-128-S1.docx]

**Table S2.** Basic statistics of grain phenotype descriptors in D-genome SHWs

| **Trait** | **Mean** | **Minimum** | **Maximum** | **Std.Dev.** | **CV(%)** | ***h^2^* (bs)** |
| --- | --- | --- | --- | --- | --- | --- |
| HArea | 0.213 | 0.164 | 0.269 | 0.018 | 8.57 | 0.86 |
| HPerim. | 2.214 | 1.775 | 2.853 | 0.163 | 7.355 | 0.83 |
| Length | 0.824 | 0.683 | 0.928 | 0.045 | 5.429 | 0.67 |
| Width | 0.328 | 0.276 | 0.381 | 0.019 | 5.883 | 0.81 |
| HRound | 0.401 | 0.327 | 0.486 | 0.03 | 7.567 | 0.71 |
| HDFE | 7.611 | 6.395 | 8.553 | 0.381 | 5.008 | 0.82 |
| Weight | 51.272 | 36.12 | 64.28 | 5.947 | 11.599 | 0.87 |
| VArea | 0.103 | 0.078 | 0.125 | 0.009 | 8.988 | 0.92 |
| VPerim. | 1.389 | 1.136 | 1.618 | 0.08 | 5.757 | 0.88 |
| Thickness | 0.332 | 0.288 | 0.376 | 0.016 | 4.817 | 0.74 |
| VRound | 0.851 | 0.768 | 0.898 | 0.021 | 2.434 | 0.78 |
| VDFE | 4.12 | 3.552 | 4.556 | 0.193 | 4.679 | 0.77 |
| Aspect ratio | 2.527 | 2.067 | 3.102 | 0.194 | 7.691 | 0.69 |
| Volume | 0.378 | 0.25 | 0.511 | 0.045 | 11.832 | 0.8 |
| FFD | 4.745 | 3.2 | 5.719 | 0.41 | 8.644 | 0.86 |
| Comp1 | 0.022 | 0.018 | 0.026 | 0.002 | 7.148 | 0.75 |
