# Supplementary figures and images for: Genome-wide association for grain morphology in synthetic hexaploid wheats using digital imaging analysis
Source: BMC Plant Biol. 2014 May 9;14:128. doi: 10.1186/1471-2229-14-128 (PMC4057600; doi:10.1186/1471-2229-14-128)

**
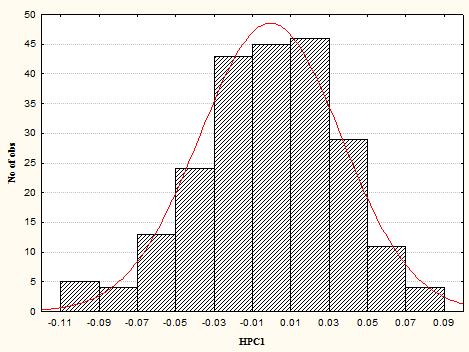

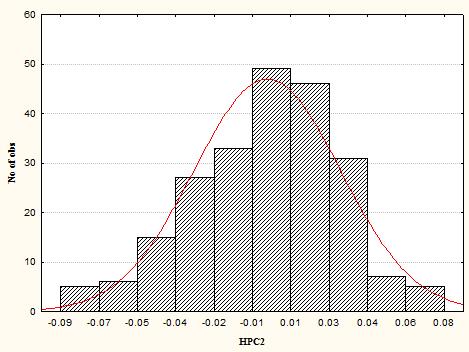

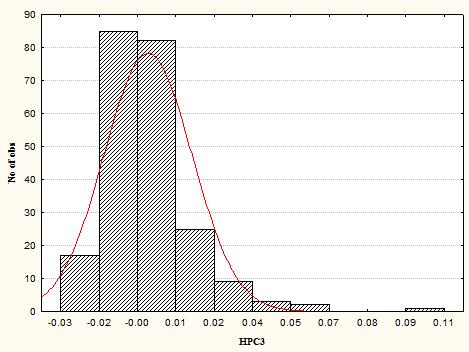

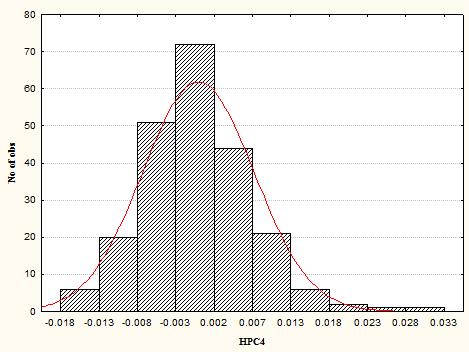

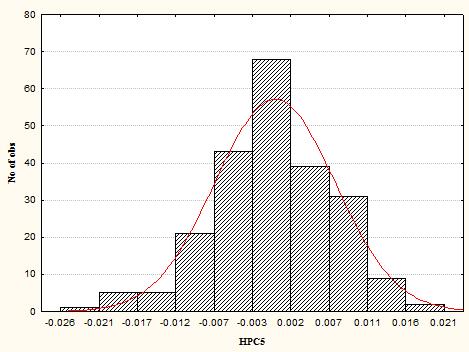

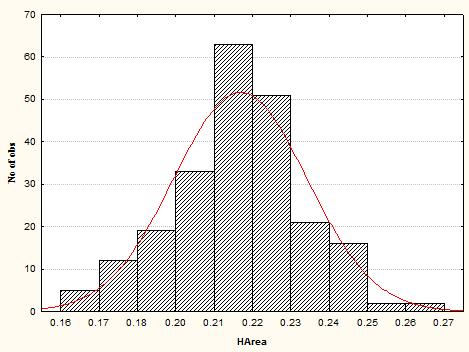

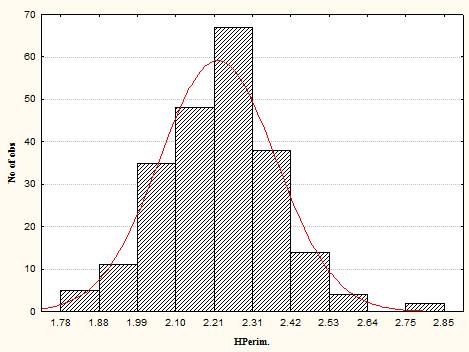

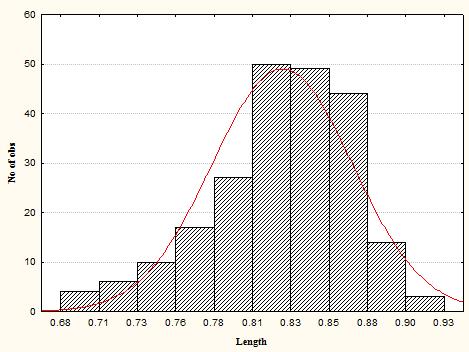

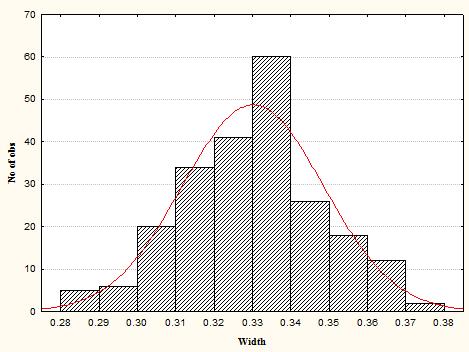

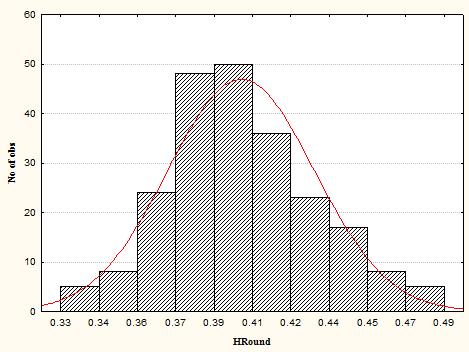

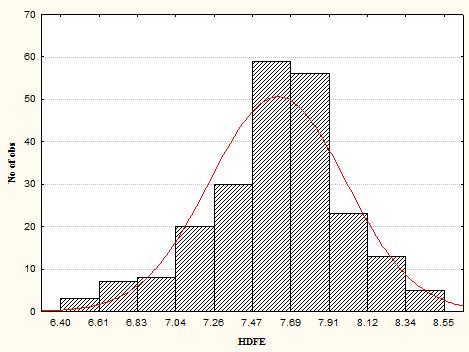

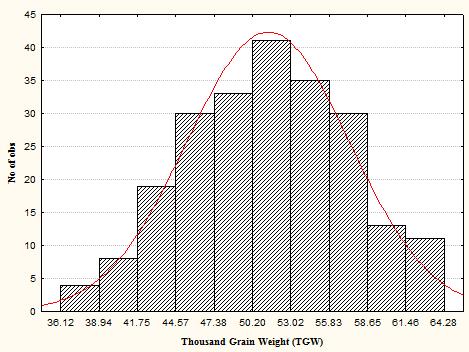

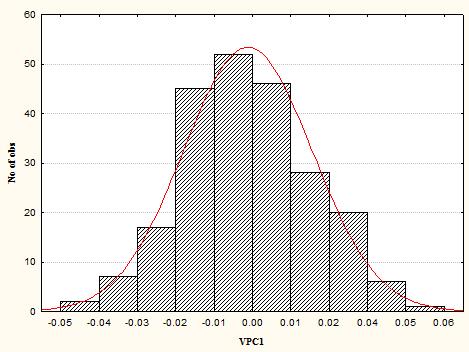

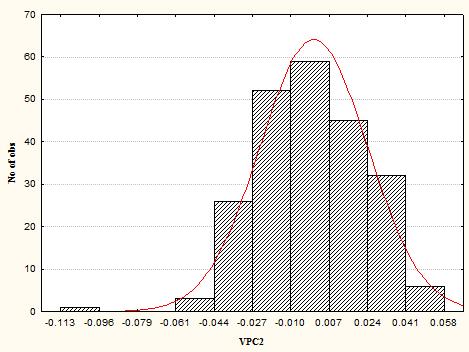

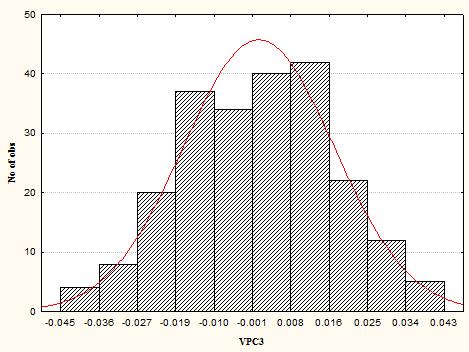

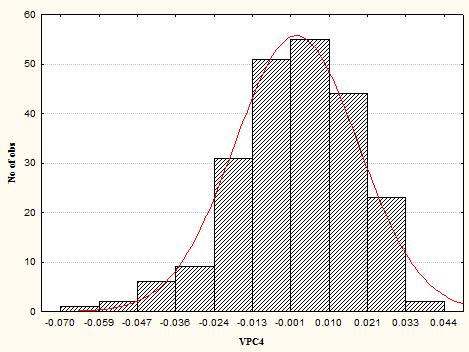

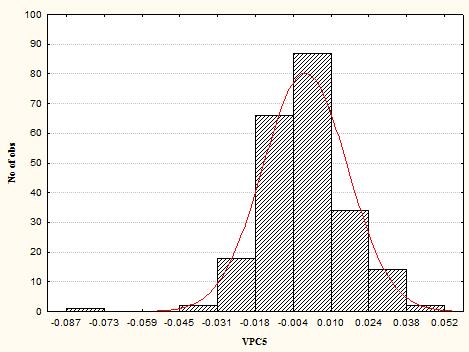

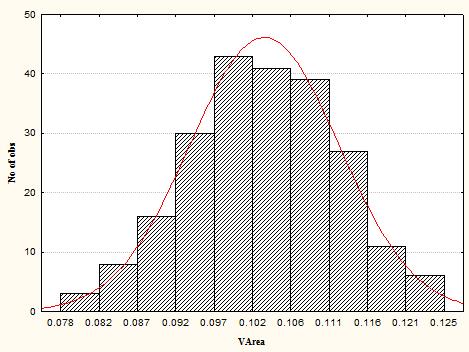

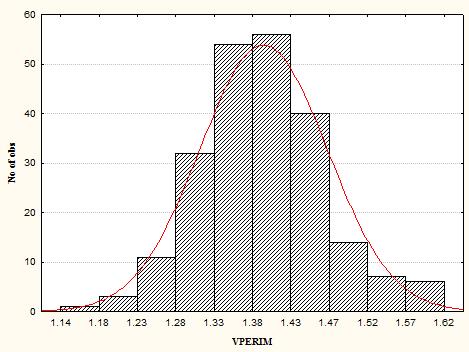

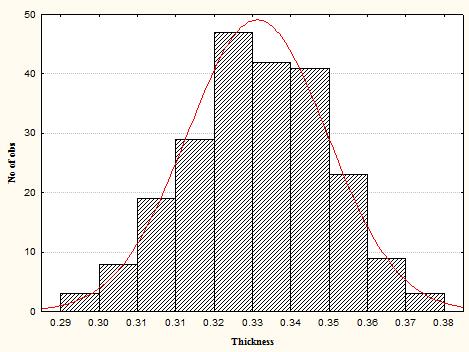

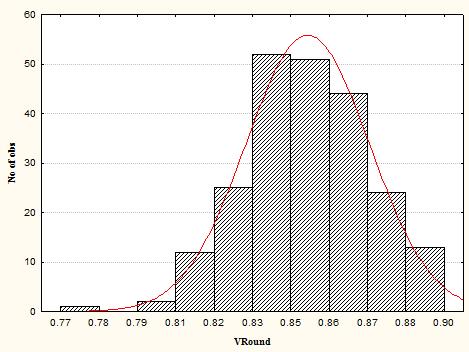

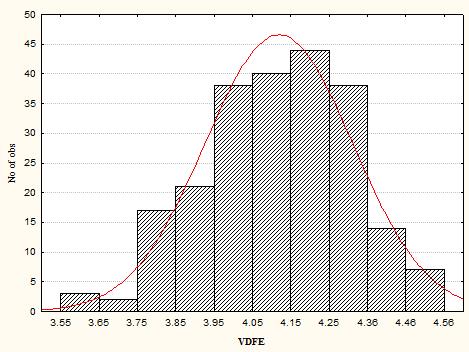

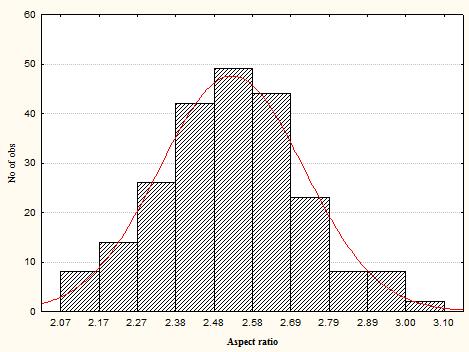

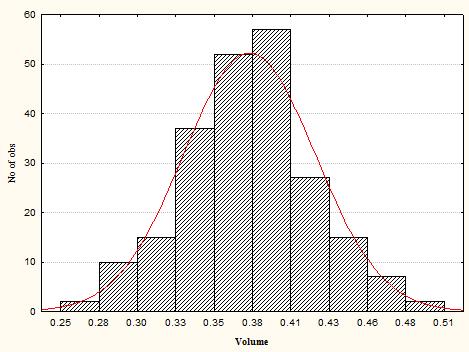

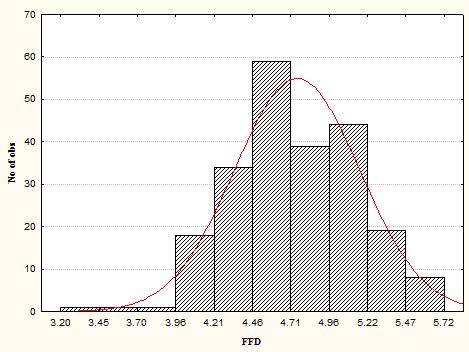

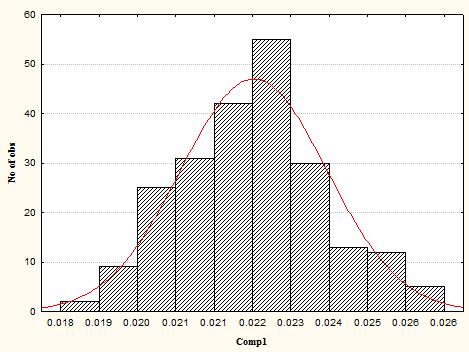

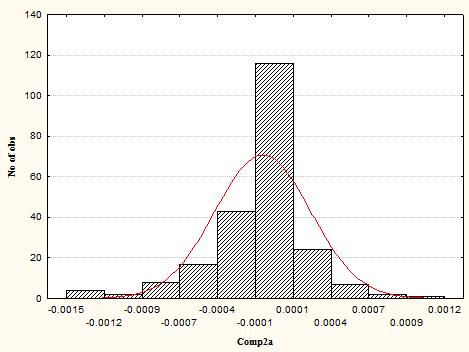

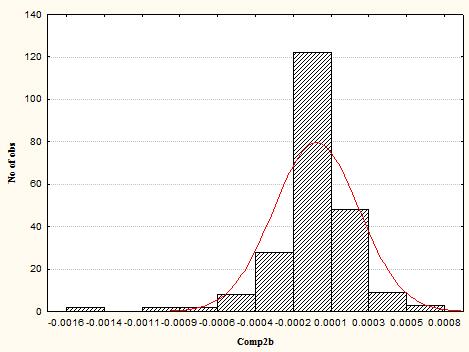

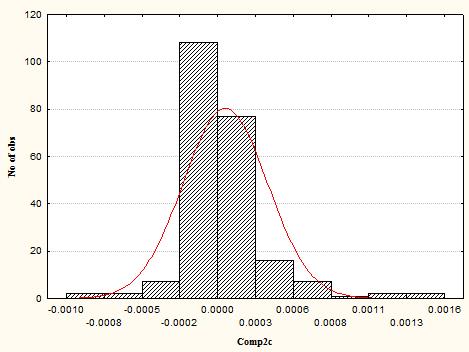
**

**Figure S1.** Frequency distribution of all traits related to grain size and shape in SHW

Supplement: Additional file 2: Figure S1 — Frequency distribution of all traits related to grain size and shape in SHWs. [file 1471-2229-14-128-S2.docx]
