# Supplementary material for: Genome-wide association for grain morphology in synthetic hexaploid wheats using digital imaging analysis
Source: BMC Plant Biol. 2014 May 9;14:128. doi: 10.1186/1471-2229-14-128 (PMC4057600; doi:10.1186/1471-2229-14-128)
Supplement: Additional file 3: Figure S5 — LD heatmap of all wheat chromosomes showing extent of pair wise linkage dis-equilibrium between DArT markers. [file 1471-2229-14-128-S3.docx]

**
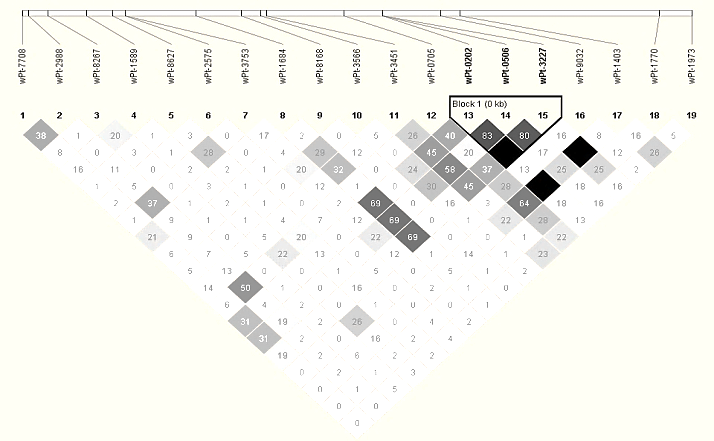

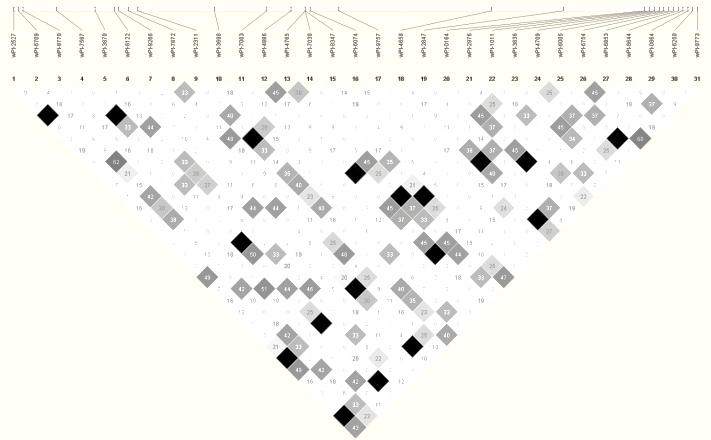

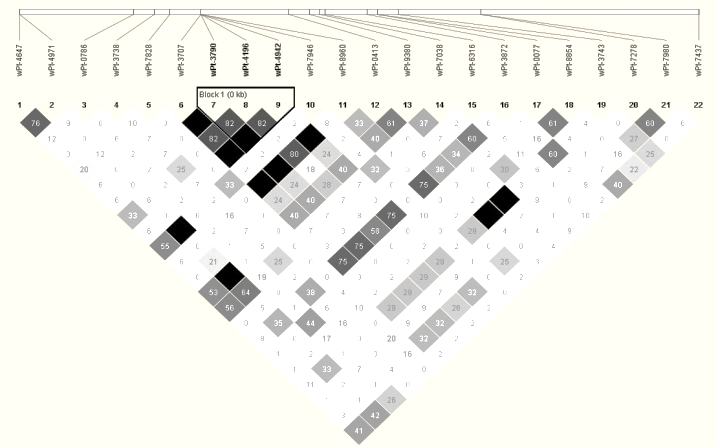

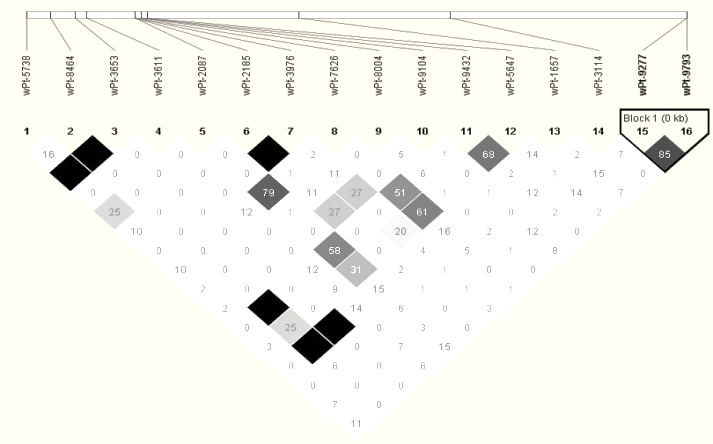

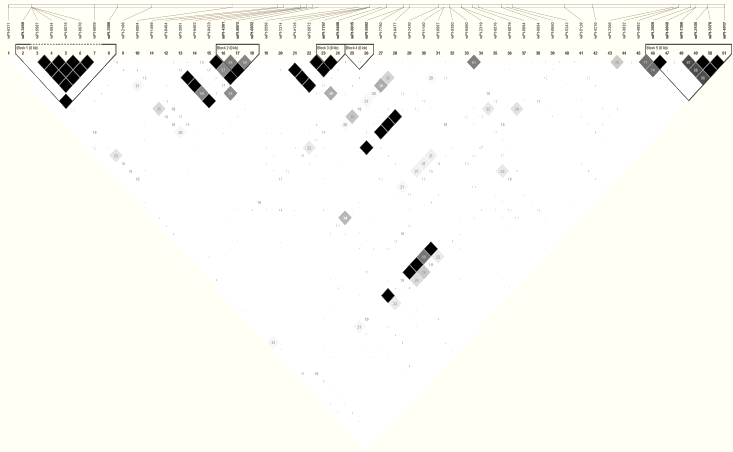

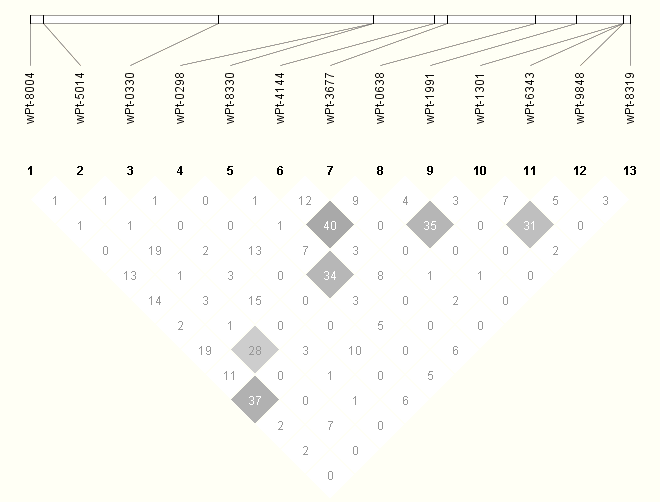

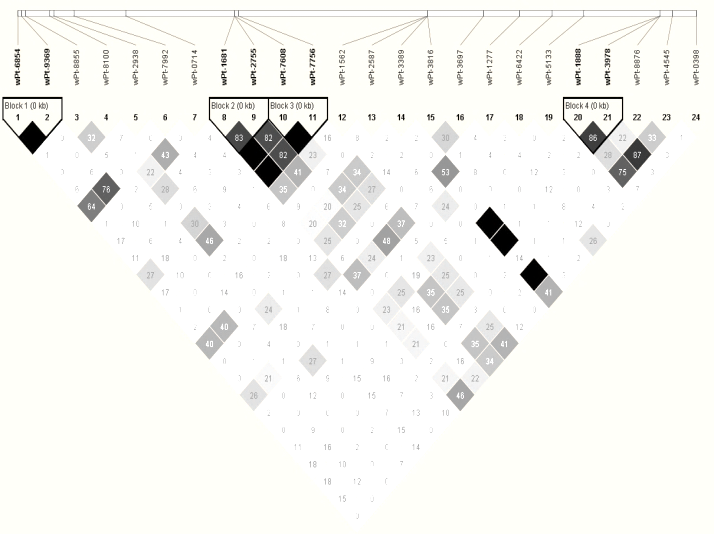

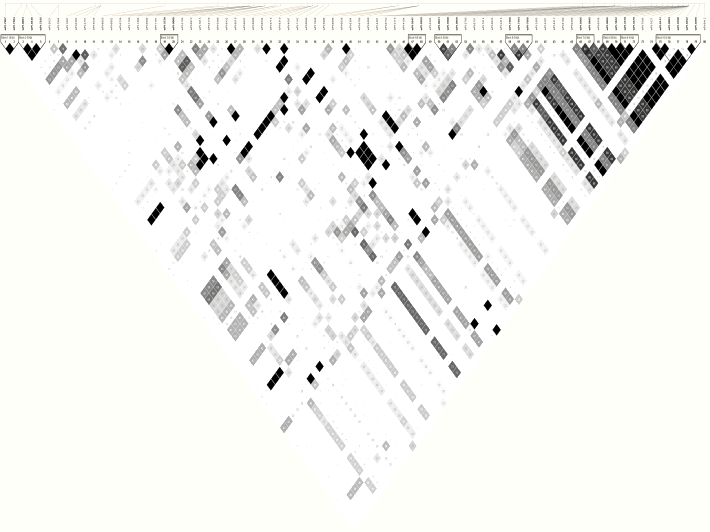

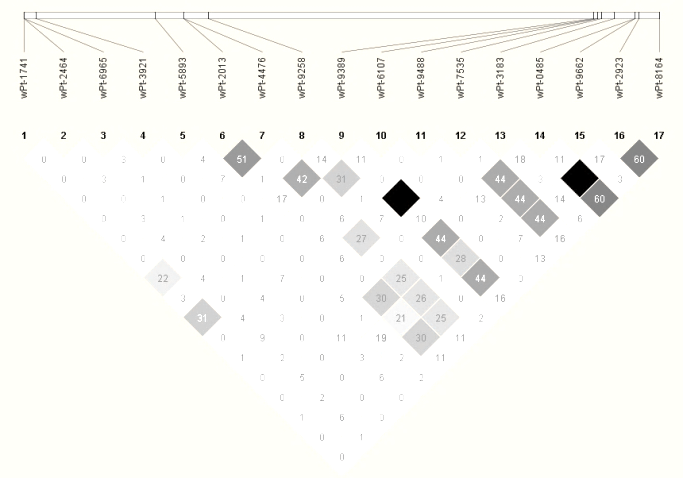

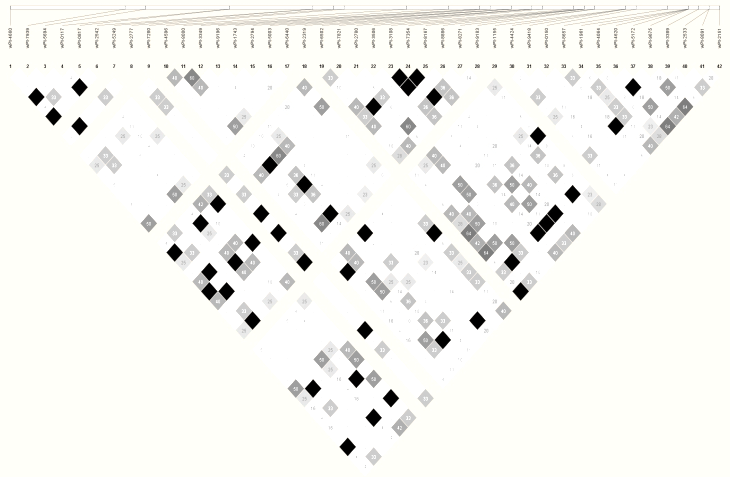

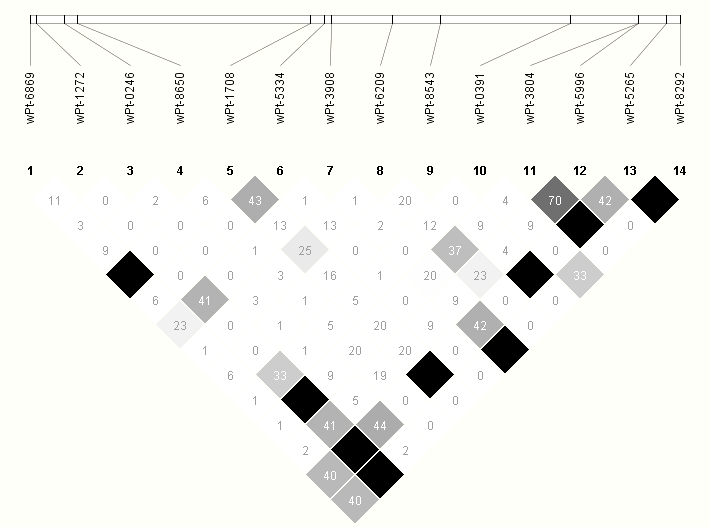

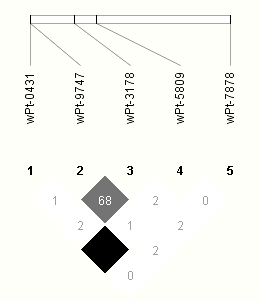

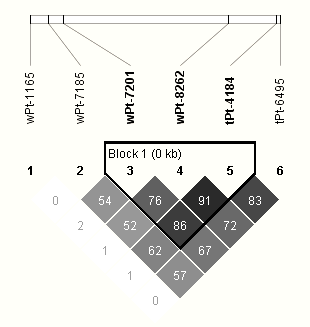

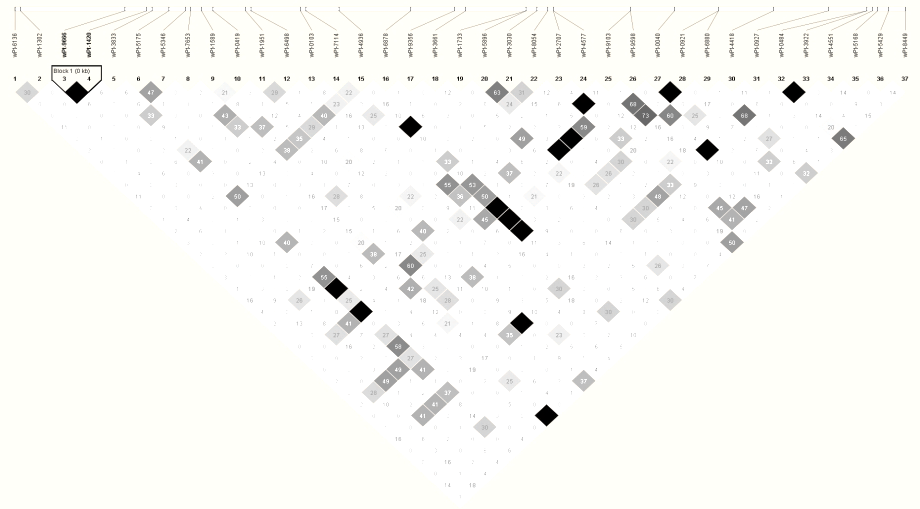

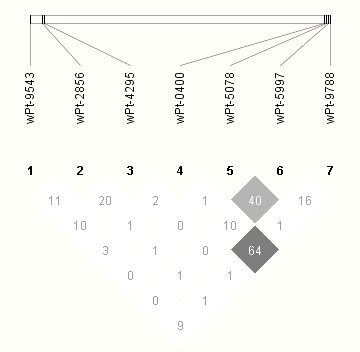

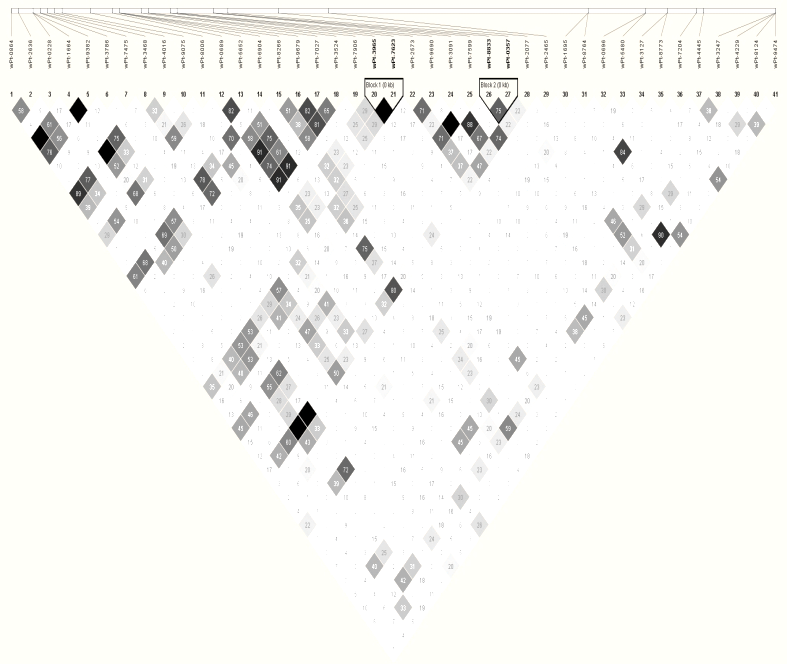

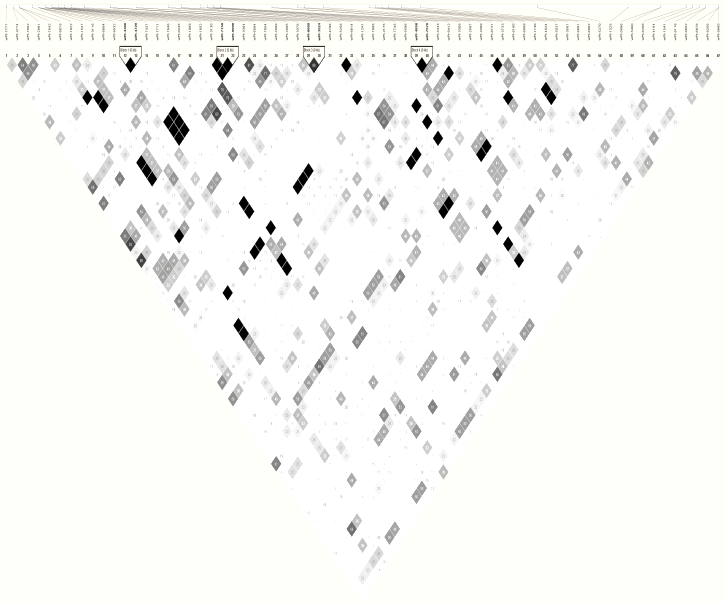

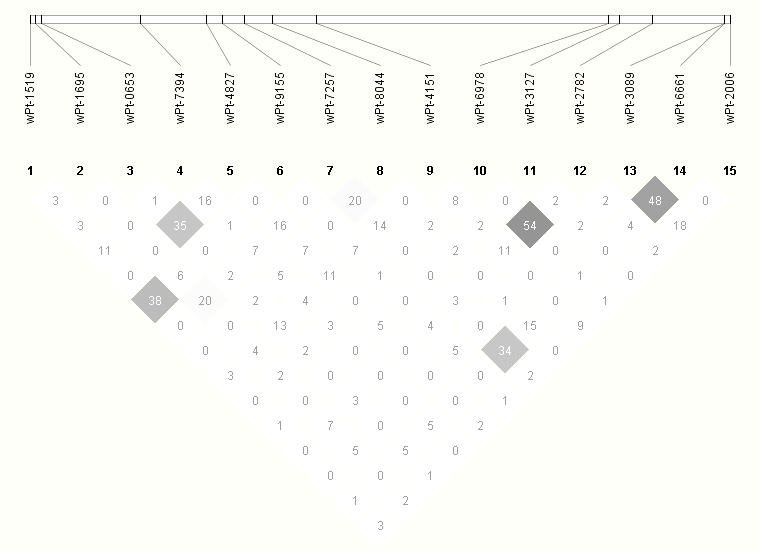

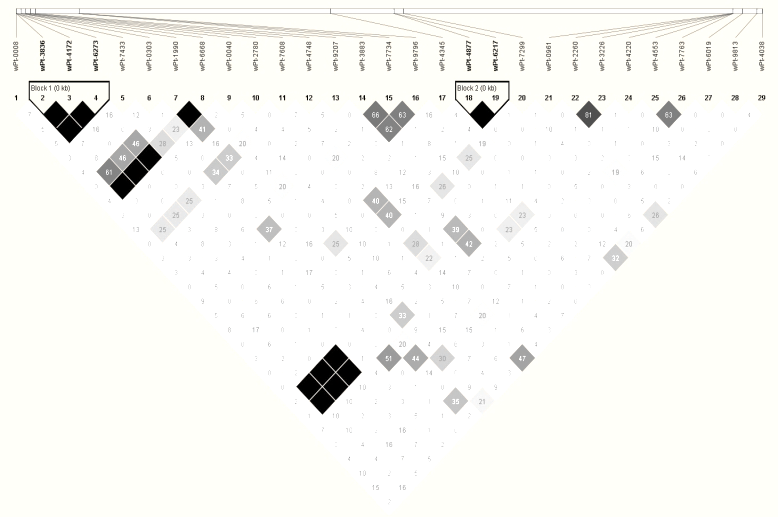

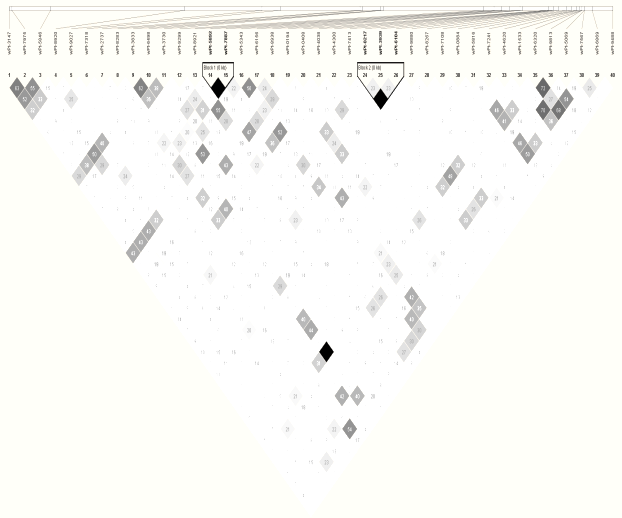
**

**
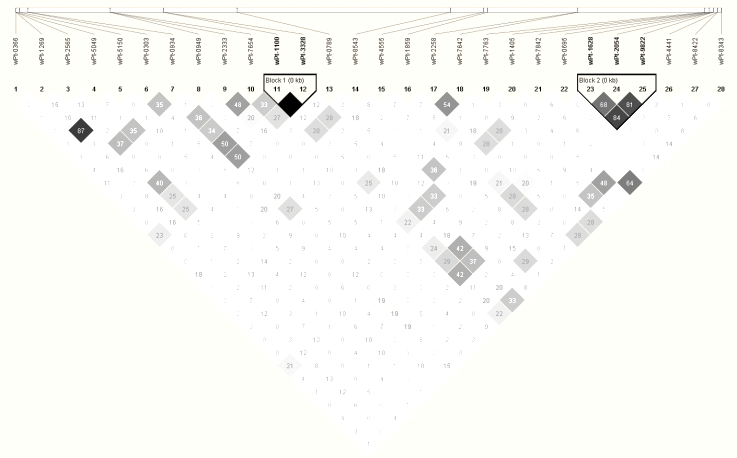
**

**Figure S5.** LD heatmap of all wheat chromosomes showing extent of pair wise linkage dis-equilibrium between DArT markers.
