# Supplementary material for: Genome-wide association for grain morphology in synthetic hexaploid wheats using digital imaging analysis
Source: BMC Plant Biol. 2014 May 9;14:128. doi: 10.1186/1471-2229-14-128 (PMC4057600; doi:10.1186/1471-2229-14-128)
Supplement: Additional file 4: Table S4 — MTAs identified for grain shape in SHWs. [file 1471-2229-14-128-S4.docx]

**Table S4.** Marker-trait associations (MTAs) for grain shape characteristics in D genome synthetic hexaploid wheats

| Trait | Marker | Chr | Pos | MAF | GLM | | | MLM | | QTL/Gene | Reference |
| --- | --- | --- | --- | --- | --- | --- | --- | --- | --- | --- | --- |
|  |  |  |  |  | | *P* values *R^2^* (%) | | *P* values *R^2^* (%) | |  |  |
| Aspect ratio | wPt-6880 | 5B | 128.94 | 0.16 | 6.75E-04 | | 0.049 | 1.29E-03 | 0.048 |  |  |
|  | TaGW2 | 6A | 36.2 |  | 0.0097 | | 0.0918 |  |  |  |  |
|  | wPt0884 | 7B | 219.72 |  |  | |  | 1.73E-03 | 0.063 |  |  |
| Comp1 | wPt-1830 | 6B | 36.12 | 0.38 | 6.30E-04 | | 0.068 |  |  |  |  |
| Comp2a | wPt-5092 | 5B | 59.8 | 0.32 | 2.93E-04 | | 0.073 | 1.77E-03 | 0.065 |  |  |
|  | wPT4418 | 5B | 138.17 |  |  | |  | 6.74E-04 | 0.058 |  |  |
|  | wPt-4016 | 6A | 7.66 | 0.42 | 6.72E-04 | | 0.088 |  |  |  |  |
|  | tPt-4887 | 6B | 33.17 | 0.18 | 9.56E-04 | | 0.059 |  |  |  |  |
|  | wPt-4858 | 6B | 36.15 | 0.24 | 9.20E-04 | | 0.051 |  |  |  |  |
|  | wPt-7576 | 6B | 36.15 | 0.24 | 9.88E-04 | | 0.051 |  |  |  |  |
|  | tPt-3506 | 6B | 36.15 | 0.18 | 2.86E-04 | | 0.07 | 1.29E-03 | 0.065 |  |  |
|  | wPt-8194 | 6B | 48.12 | 0.30 | 5.56E-04 | | 0.063 |  |  |  |  |
|  | wPt-7846 | 6B | 48.12 | 0.29 | 2.62E-04 | | 0.074 | 1.74E-03 | 0.068 |  |  |
|  | wPt-9971 | 6B | 50.46 | 0.31 | 2.21E-04 | | 0.072 | 1.59E-03 | 0.063 |  |  |
|  | wPt-9665 | 7B | 149.49 | 0.34 | 8.20E-04 | | 0.061 |  |  |  |  |
|  | wPt-8981 | 7B | 149.49 | 0.33 | 3.19E-04 | | 0.07 | 1.80E-03 | 0.064 |  |  |
|  | wPt-8312 | 7B | 152.31 | 0.17 | 4.61E-04 | | 0.065 |  |  |  |  |
|  | tPt-7362 | 7B | 196.42 | 0.23 | 9.26E-04 | | 0.058 |  |  |  |  |
|  | wPt-4298 | 7B | 196.42 | 0.13 | 2.21E-04 | | 0.075 | 1.10E-03 | 0.073 |  |  |
| Comp2b | wPt-5249 | 4A | 68.11 | 0.36 | 6.07E-04 | | 0.09 | 1.58E-03 | 0.09 |  |  |
| Comp2c | wPt-2706 | 1B | 28.53 | 0.21 | 6.99E-04 | | 0.068 |  |  |  |  |
|  | wPt-7242 | 1B | 29.6 | 0.21 | 8.96E-04 | | 0.068 |  |  |  |  |
|  | wPt-5385 | 1B | 30.97 | 0.23 | 8.45E-04 | | 0.063 |  |  |  |  |
|  | wPt-6047 | 3B | 63.18 | 0.39 | 9.60E-04 | | 0.054 |  |  |  |  |
|  | wPt-5092 | 5B | 59.8 |  | 1.97E-04 | | 0.083 |  |  |  |  |
|  | wPt-7906 | 6A | 20.49 | 0.09 | 1.11E-03 | | 0.056 |  |  |  |  |
|  | tPt-4209 | 6A | 48.82 | 0.39 | 1.05E-04 | | 0.087 | 5.92E-04 | 0.062 |  |  |
|  | wPt-1730 | 6B | 62.51 | 0.31 | 2.24E-04 | | 0.066 |  |  |  |  |
| HDFE | wPt-6530 | 1A | 84.27 |  | 6.65E-04 | | 0.058 |  |  |  |  |
|  | wPt-3976 | 2A | 18.83 | 0.17 |  | |  | 7.14E-04 | 0.084 |  |  |
|  | wPt-2185 | 2A | 18.83 | 0.17 |  | |  | 7.53E-04 | 0.082 |  |  |
|  | wPt-0298 | 2D | 66.69 | 0.31 | 7.10E-04 | | 0.079 |  |  |  |  |
|  | wPt-8356 | 3B | 45.21 |  | 6.49E-06 | | 0.102 | 3.06E-04 | 0.072 |  |  |
|  | wPt-6239 | 3B | 49.01 | 0.40 | 1.16E-03 | | 0.073 |  |  |  |  |
|  | wPt-1940 | 3B | 68.58 |  | 8.66E-04 | | 0.05 |  |  |  |  |
|  | wPt-5249 | 4A | 68.11 |  | 7.04E-04 | | 0.056 |  |  |  |  |
|  | tPt-4209 | 6A | 48.82 |  | 2.51E-04 | | 0.067 |  |  |  |  |
| HPC1 | wPt-2222 | 2A | 10.84 | 0.15 | 4.48E-04 | | 0.068 |  |  |  |  |
|  | wPt-1615 | 2A | 95.09 | 0.25 | 5.50E-05 | | 0.084 |  |  |  |  |
|  | wPt-2858 | 2A | 95.09 | 0.25 | 3.05E-05 | | 0.09 |  |  |  |  |
|  | wPt-1294 | 2B | 78.89 | 0.19 | 5.64E-04 | | 0.062 |  |  |  |  |
|  | wPt-2644 | 2D | 70.93 | 0.38 | 3.20E-04 | | 0.074 |  |  |  |  |
|  | wPt-1940 | 3B | 68.58 |  |  | |  | 7.49E-04 | 0.057 |  |  |
|  | tPt-9048 | 6A | 89.77 | 0.37 | 9.06E-04 | | 0.063 |  |  |  |  |
|  | wPt-5333 | 6B | 37.6 | 0.45 | 6.71E-04 | | 0.055 |  |  |  |  |
|  | wPt-3309 | 6B | 38.28 | 0.41 | 3.77E-04 | | 0.065 |  |  |  |  |
|  | wPt-9971 | 6B | 50.46 |  | 4.82E-04 | | 0.064 |  |  |  |  |
| HPC2 | wPt-6880 | 5B | 128.94 |  | 2.03E-04 | | 0.058 | 3.85E-04 | 0.068 |  |  |
| HPC3 | wPt-1991 | 2D | 96.63 | 0.23 | 1.01E-03 | | 0.054 | 6.73E-04 | 0.061 |  |  |
| HPC4 | wPt-8463 | 3D | 53.86 | 0.28 | 6.32E-04 | | 0.067 | 8.29E-04 | 0.069 |  |  |
|  | wPt-4418 | 5B | 138.18 |  | 1.57E-04 | | 0.097 |  |  |  |  |
| HPC5 | wPt-3477 | 1B | 9.16 | 0.17 | 1.10E-03 | | 0.056 |  |  |  |  |
|  | wPt-3561 | 2B | 51.43 | 0.28 | 7.21E-04 | | 0.055 |  |  |  |  |
|  | wPt-1940 | 3B | 68.58 |  | 1.20E-05 | | 0.088 | 1.63E-05 | 0.094 |  |  |
|  | wPt-3810 | 4A | 59.66 | 0.37 | 8.23E-04 | | 0.062 |  |  |  |  |
| Hperim | wPt-2185 | 2A | 18.83 |  |  | |  | 1.94E-03 | 0.069 |  |  |
|  | wPt-9277 | 2A | 109.43 |  | 7.03E-04 | | 0.076 |  |  |  |  |
|  | wPt-9793 | 2A | 109.43 |  | 4.89E-04 | | 0.084 |  |  |  |  |
|  | wPt-8356 | 3B | 45.21 |  | 1.14E-05 | | 0.096 |  |  |  |  |
|  | wPt-4620 | 4A | 104.6 | 0.25 | 1.17E-03 | | 0.048 |  |  |  |  |
|  | wPt-7599 | 6A | 29.44 | 0.15 | 9.66E-04 | | 0.071 |  |  |  |  |
| HRound | wPt-6880 | 5B | 128.94 |  | 1.14E-04 | | 0.062 | 2.25E-04 | 0.063 |  |  |
|  | wPt-4706 | 6B | 21.21 | 0.23 | 8.94E-04 | | 0.068 |  |  |  |  |
| VDFE | wPt-4886 | 1A | 60.73 | 0.47 | 8.63E-04 | | 0.054 |  |  |  |  |
|  | wPt-5256 | 6B | 25.79 | 0.20 | 7.89E-04 | | 0.054 |  |  |  |  |
|  | wPt-8814 | 6B | 25.79 | 0.20 | 2.86E-04 | | 0.064 | 8.53E-04 | 0.058 |  |  |
|  | tPt-4887 | 6B | 33.17 |  | 9.95E-04 | | 0.062 |  |  |  |  |
|  | tPt-3506 | 6B | 36.15 |  | 1.17E-03 | | 0.059 |  |  |  |  |
| VPC1 | wPt-8915 | 3B | 58.38 |  | 8.53E-06 | | 0.111 | 1.15E-05 | 0.126 |  |  |
|  | wPt-5857 | 4A | 68.11 | 0.40 | 1.04E-03 | | 0.062 |  |  |  |  |
| VPC2 | wPt-4886 | 1A | 60.73 |  | 8.67E-05 | | 0.068 | 5.18E-04 | 0.059 |  |  |
|  | wPt-7992 | 3A | 58.95 |  | 9.42E-04 | | 0.052 |  |  |  |  |
|  | wPt-1655 | 3A | 60.25 | 0.33 | 4.81E-04 | | 0.063 |  |  |  |  |
|  | wPt-5390 | 3B | 58.38 | 0.19 | 3.53E-04 | | 0.058 | 1.36E-03 | 0.054 |  |  |
|  | wPt-1940 | 3B | 68.58 |  | 9.78E-05 | | 0.068 | 8.52E-05 | 0.077 |  |  |
| VPC3 | wPt-5647 | 2A | 20.09 | 0.44 | 9.35E-04 | | 0.077 |  |  |  |  |
|  | wPt-1657 | 2A | 45.72 | 0.23 | 8.00E-04 | | 0.081 |  |  |  |  |
|  | wPt-6311 | 2B | 0 | 0.15 | 6.93E-04 | | 0.083 |  |  |  |  |
|  | wPt-9859 | 2B | 14.6 | 0.14 | 1.95E-04 | | 0.093 |  |  |  |  |
|  | wPt-1489 | 2B | 46.59 | 0.08 | 6.56E-04 | | 0.078 |  |  |  |  |
|  | wPt-9423 | 2B | 51.63 | 0.09 | 6.74E-04 | | 0.08 |  |  |  |  |
|  | wPt-9402 | 2B | 51.63 | 0.07 | 1.32E-04 | | 0.1 | 1.02E-03 | 0.082 |  |  |
|  | wPt-4301 | 2B | 52.3 | 0.19 | 1.05E-03 | | 0.075 |  |  |  |  |
|  | wPt-8072 | 2B | 53.39 | 0.08 | 6.77E-04 | | 0.08 |  |  |  |  |
|  | wPt-3389 | 3A | 146.74 | 0.06 | 9.67E-05 | | 0.101 | 4.65E-04 | 0.089 |  |  |
|  | wPt-2533 | 4A | 106.95 | 0.07 | 5.20E-05 | | 0.11 | 2.91E-04 | 0.096 |  |  |
|  | wPt-8091 | 4A | 106.95 | 0.07 | 1.30E-04 | | 0.098 | 5.56E-04 | 0.092 |  |  |
|  | wPt-2707 | 5B | 98.54 | 0.16 | 1.15E-03 | | 0.073 |  |  |  |  |
|  | wPt-4577 | 5B | 98.54 | 0.17 | 1.10E-03 | | 0.072 |  |  |  |  |
|  | TaSAP | 7A | 54.1 |  | 0.0193 | | 0.0632 |  |  |  |  |
| VPC4 | wPt-9757 | 1A | 70.87 | 0.22 | 8.57E-04 | | 0.078 | 8.32E-04 | 0.084 |  |  |
|  | wPt-6576 | 2B | 79.07 | 0.32 | 6.83E-05 | | 0.111 | 7.83E-05 | 0.118 | *Ppd-B1* |  |
|  | wPt-4892 | 2B | 116.73 | 0.40 |  | |  | 1.54E-03 | 0.075 |  |  |
|  | wPt-2929 | 2B | 118.58 | 0.41 |  | |  | 1.25E-03 | 0.079 |  |  |
|  | wPt-1655 | 3A | 60.25 |  | 6.76E-04 | | 0.059 |  |  |  |  |
| VPerim | wPt-2858 | 2A | 95.09 |  | 7.99E-04 | | 0.064 |  |  |  |  |
|  | wPt-1615 | 2D | 112.64 |  | 5.76E-04 | | 0.067 |  |  | MQTL20 | Zhang et al (2010) |
|  | wPt-2858 | 2D | 112.64 |  | 7.99E-04 | | 0.064 |  |  | MQTL20 | Zhang et al (2010) |
|  | TaSAP | 7A | 54.1 |  | 0.0308 | | 0.0579 |  |  |  |  |
| VRound | wPt-4660 | 4A | 39.75 |  | 1.00E-03 | | 0.075 |  |  |  |  |
|  | wPt-5497 | 4B | 57.9 | 0.21 | 2.79E-04 | | 0.077 |  |  |  |  |
|  | wPt-6123 | 4B | 57.9 | 0.22 | 6.14E-04 | | 0.065 |  |  |  |  |
|  | wPt-5334 | 4B | 58.5 | 0.24 | 2.29E-04 | | 0.071 |  |  |  |  |
|  | wPt-1922 | 6B | 9.8 | 0.17 | 6.38E-04 | | 0.064 |  |  |  |  |
|  | wPt-4386 | 6B | 11.51 | 0.38 | 5.62E-04 | | 0.056 |  |  |  |  |
|  | wPt-7150 | 6B | 11.51 | 0.19 | 5.62E-04 | | 0.056 |  |  |  |  |
|  | wPt-4720 | 6B | 11.72 | 0.19 | 5.15E-04 | | 0.057 |  |  |  |  |
|  | wPt-3130 | 6B | 12.71 | 0.19 | 5.51E-04 | | 0.056 |  |  |  |  |
|  | wPt-8563 | 6B | 12.71 | 0.17 | 6.69E-04 | | 0.065 |  |  |  |  |
|  | wPt-9990 | 6B | 12.71 | 0.19 | 5.62E-04 | | 0.056 |  |  |  |  |
|  | wPt-3328 | 7D | 37.25 | 0.16 | 6.70E-04 | | 0.081 | 6.83E-04 | 0.086 |  |  |
|  | wPt-1100 | 7D | 37.25 | 0.17 | 7.33E-04 | | 0.08 | 7.03E-04 | 0.085 |  |  |
