# Supplementary material for: Genome-wide association for grain morphology in synthetic hexaploid wheats using digital imaging analysis
Source: BMC Plant Biol. 2014 May 9;14:128. doi: 10.1186/1471-2229-14-128 (PMC4057600; doi:10.1186/1471-2229-14-128)
Supplement: Additional file 5: Figure S6 — DArT consensus linkage map (Detering et al. [39]) of chromosomes showing marker-traits associations for grain size and shape in synthetic hexaploids wheat. MTAs are projected as different color solid bars for which legend is given at the end of figure. [file 1471-2229-14-128-S5.docx]

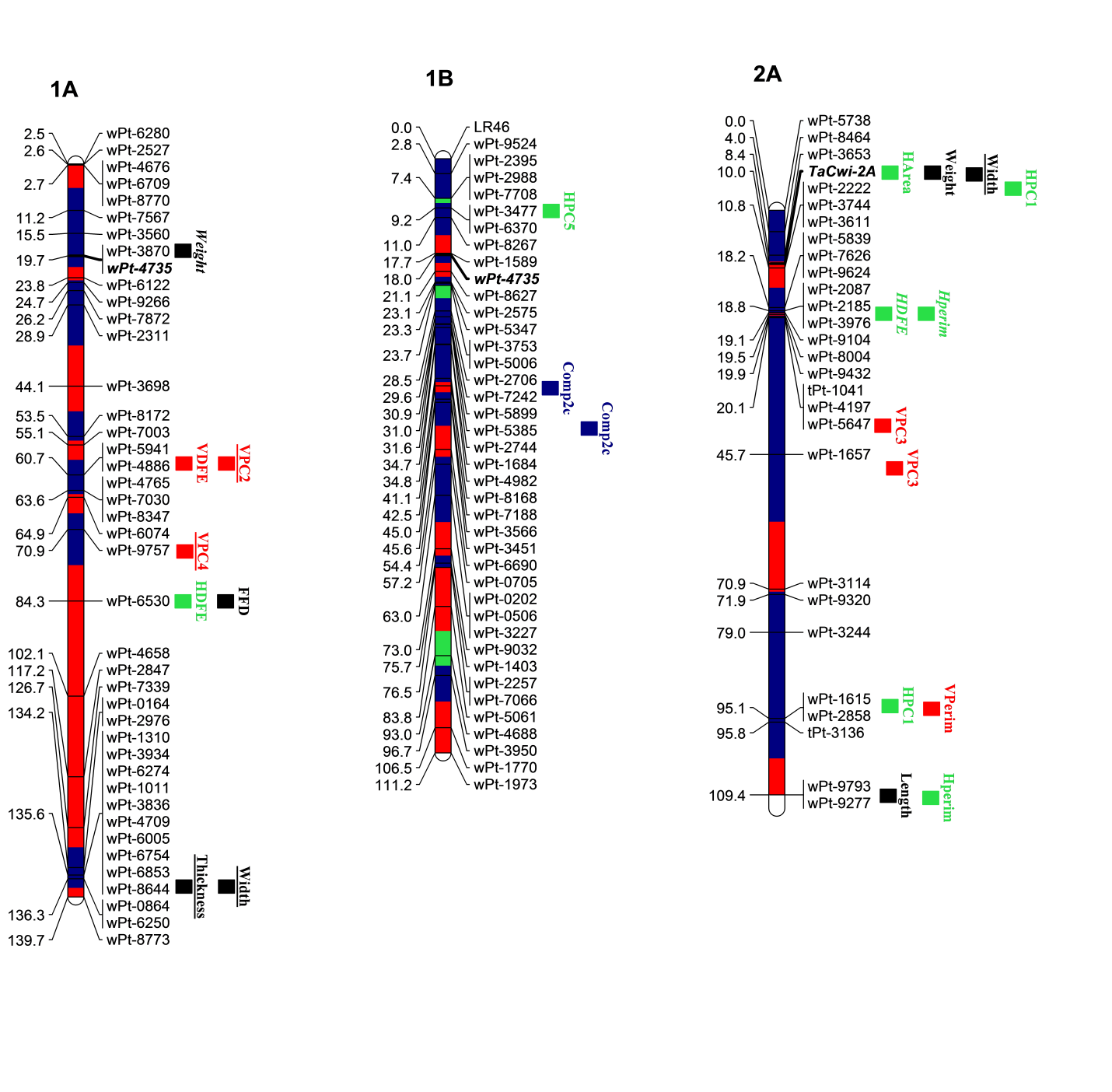

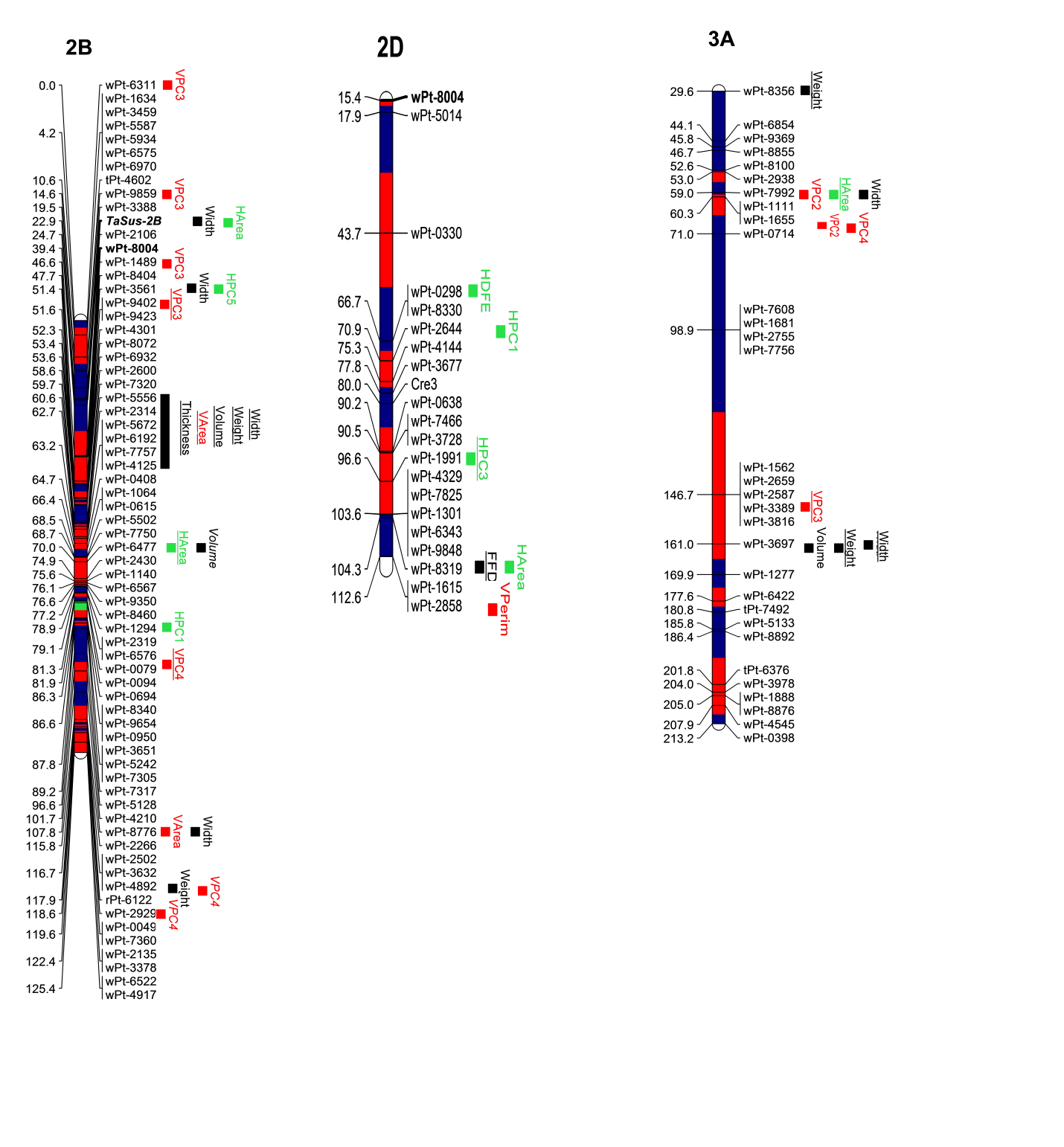

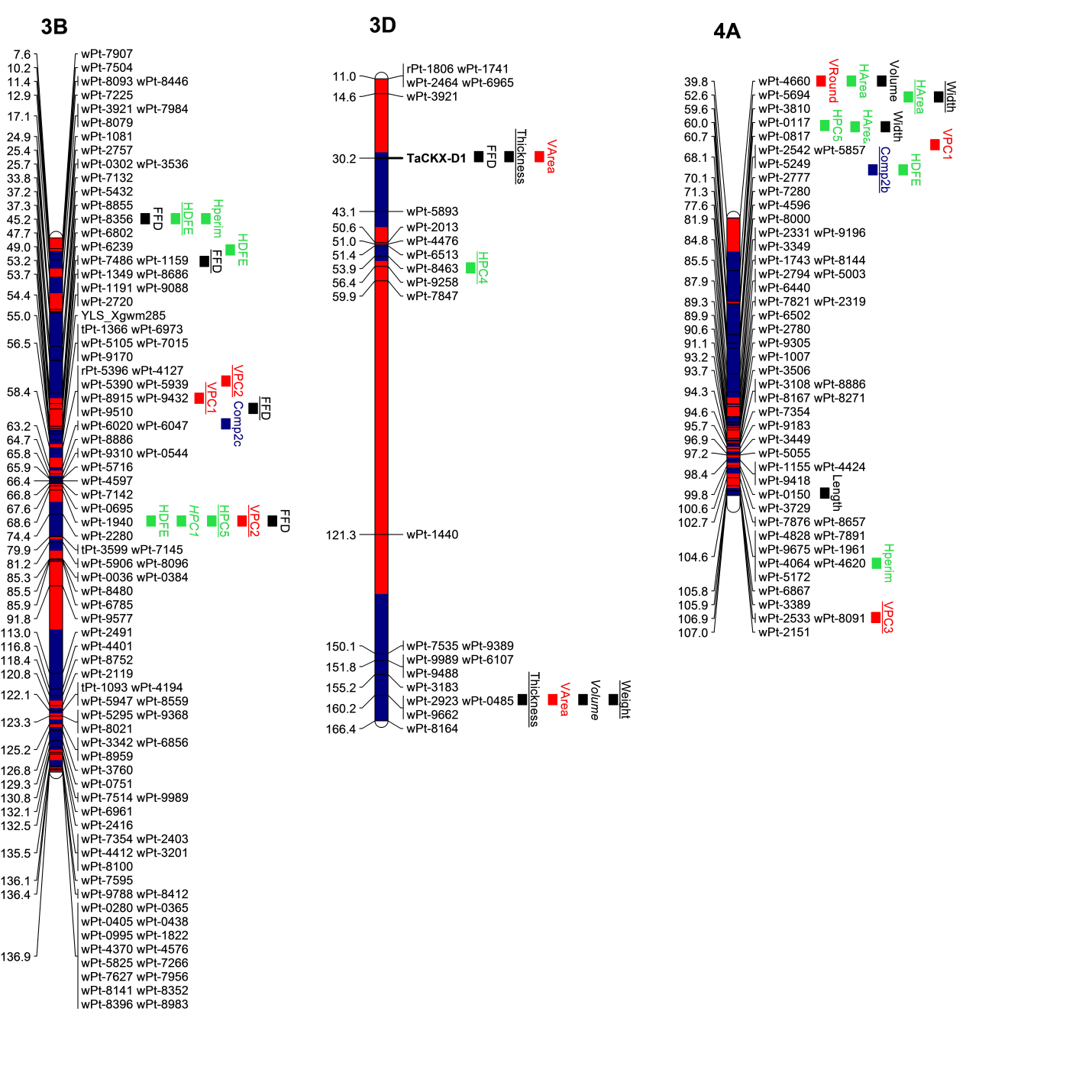

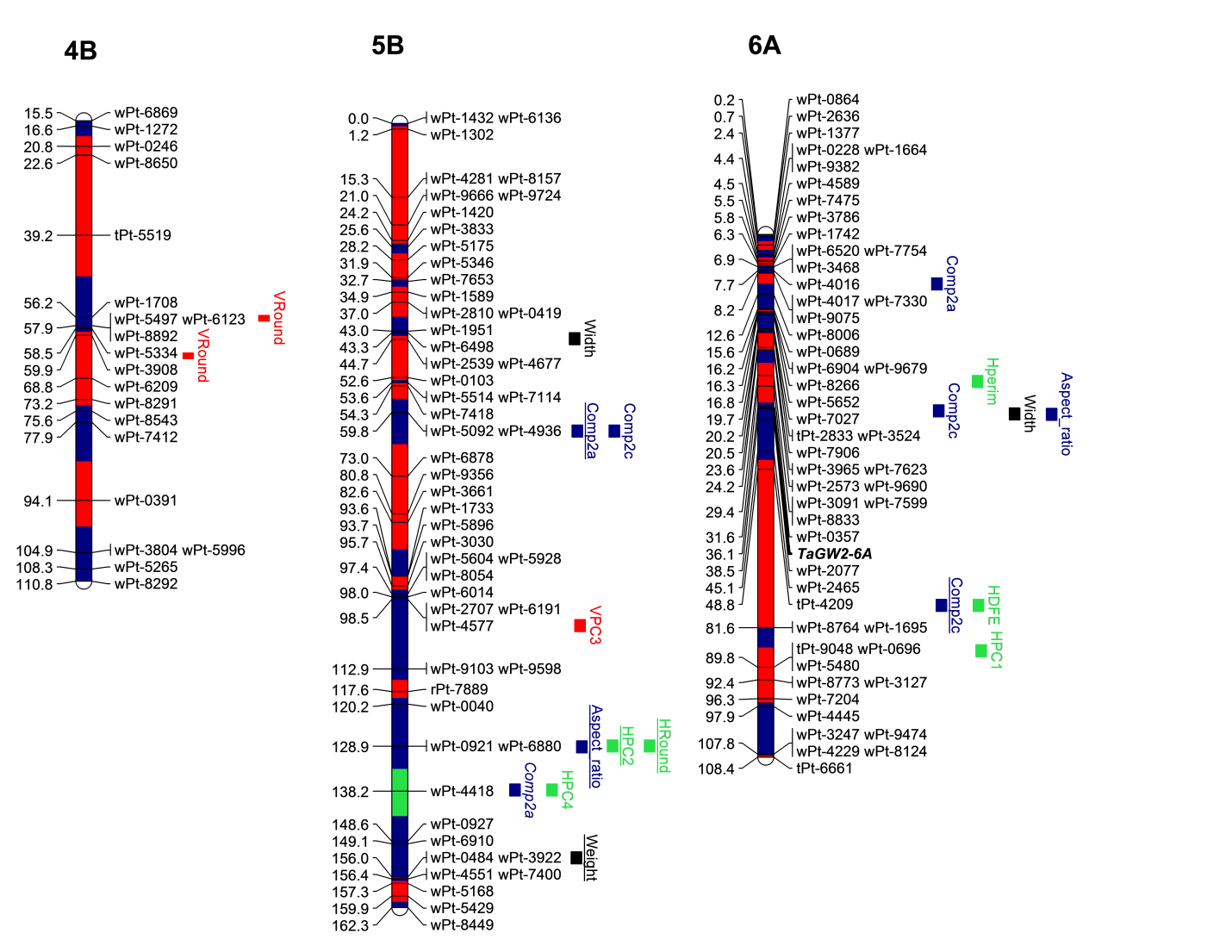

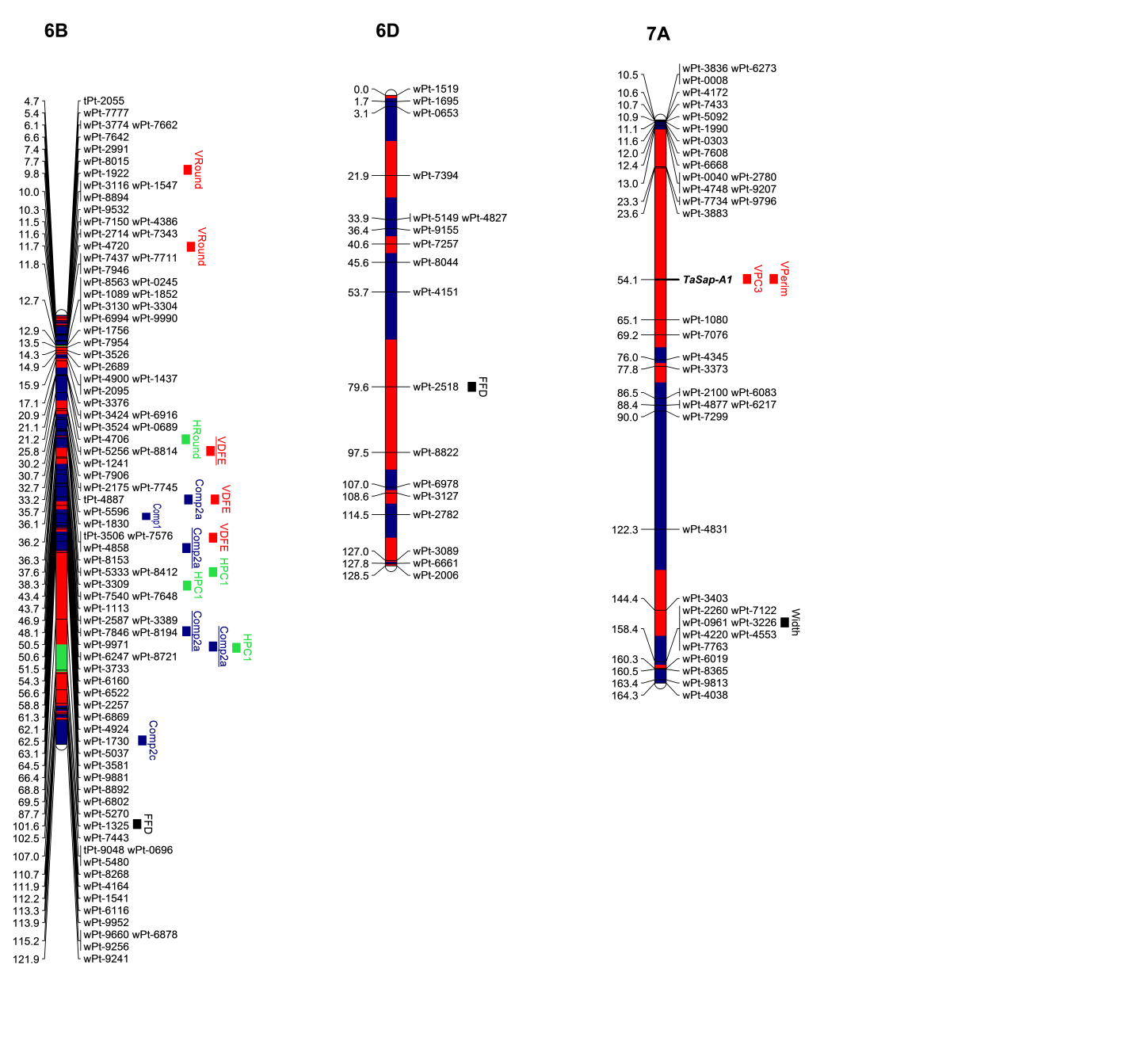

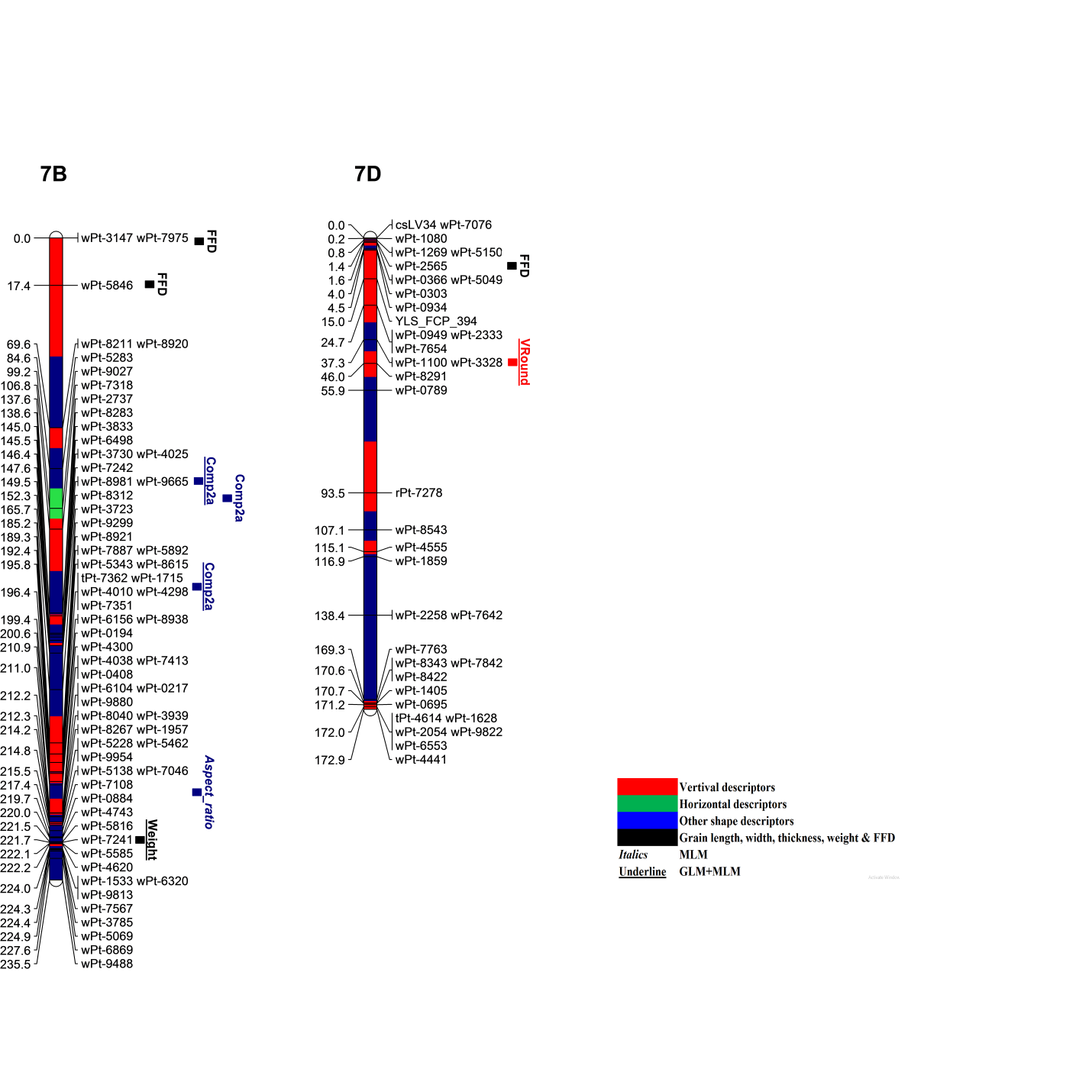


**Figure S6**: DArT consensus linkage map (Detering et al. [39]) of chromosomes showing marker-traits associations for grain size and shape in synthetic hexaploids wheat. MTAs are projected as different color solid bars for which legend is given at the end of figure
