# Supplementary material for: Genome-wide association for grain morphology in synthetic hexaploid wheats using digital imaging analysis
Source: BMC Plant Biol. 2014 May 9;14:128. doi: 10.1186/1471-2229-14-128 (PMC4057600; doi:10.1186/1471-2229-14-128)
Supplement: Additional file 6: Table S5 — Functional analysis of DArT associated with grain phenotype. [file 1471-2229-14-128-S6.docx]

**Table S5.** Functional analysis of DArT associated with grain phenotype

| DArT | Trait | GO | Seq length | Hit ACC | E-Value | Similarity | Alignment length | Positives |
| --- | --- | --- | --- | --- | --- | --- | --- | --- |
| wPt-4660 | HREA, VOL | disease resistance protein rga4 | 802 | EMS61785 | 1.03E-135 | 97 | 218 | 212 |
| wPt-2533 | VPC3 | hypothetical protein TRIUR3_19950 | 721 | EMS45218 | 3.69E-126 | 87 | 242 | 211 |
| wPt-8091 | VPC3 | hypothetical protein TRIUR3_19950 | 721 | EMS45218 | 7.41E-123 | 85 | 242 | 208 |
| wPt-3389 | VPC3 | retrotransposon ty1-copia subclass | 811 | EEE51974 | 3.67E-107 | 78 | 261 | 204 |
| wPt-9423 | VPC3 | serine threonine-protein kinase ctr1 | 767 | EMT33251 | 7.21E-91 | 80 | 204 | 165 |
| wPt-9402 | VPC3 | ribonuclease h protein at1g65750-like | 652 | EMT33251 | 2.96E-90 | 80 | 204 | 164 |
| wPt-1489 | VPC3 | serine threonine-protein kinase ctr1 | 749 | EMT33251 | 7.49E-90 | 80 | 205 | 166 |
| wPt-8072 | VPC3 | serine threonine-protein kinase ctr1 | 740 | EMT33251 | 2.81E-88 | 80 | 205 | 164 |
| wPt-1615 | VPERIM | hypothetical protein F775_00070 | 366 | EMT01391 | 1.07E-67 | 95 | 122 | 116 |
| wPt-4892 | VPC4 | ribonuclease h protein at1g65750-like | 855 | EMS51173 | 1.51E-59 | 67 | 198 | 133 |
| wPt-3477 | HPC5 | transferase family protein | 582 | EMS63107 | 2.01E-51 | 95 | 90 | 86 |
| wPt-6477 | HAREA, VOL | nucleolar protein expressed | 934 | EMS65034 | 2.47E-50 | 100 | 69 | 69 |
| wPt-3226 | VMAJOR | transposon mutator sub-class | 917 | XP_003565557 | 6.59E-47 | 67 | 177 | 120 |
| wPt-8194 | COMP2A | hypothetical protein F775_20043 | 575 | EMT03188 | 1.01E-44 | 87 | 97 | 85 |
| wPt-7241 | WEIGHT | hypothetical protein F775_18593 | 445 | EMT20614 | 2.39E-42 | 67 | 152 | 103 |
| wPt-5846 | FFD | rp3 protein | 992 | NP_001055149 | 9.66E-34 | 61 | 213 | 131 |
| wPt-5390 | VPC2 | retrotransposon unclassified | 614 | CAH66715 | 2.30E-31 | 62 | 169 | 105 |
| wPt-8319 | HAREA | hypothetical protein F775_22449 | 882 | EMT10484 | 8.49E-31 | 100 | 39 | 39 |
| wPt-8915 | FFD | predicted protein | 501 | BAJ87930 | 9.77E-29 | 92 | 67 | 62 |
