# Supplementary material for: Genome-wide association for grain morphology in synthetic hexaploid wheats using digital imaging analysis
Source: BMC Plant Biol. 2014 May 9;14:128. doi: 10.1186/1471-2229-14-128 (PMC4057600; doi:10.1186/1471-2229-14-128)
Supplement: Additional file 8: Figure S2 — Upper row: Horizontal images of synthetic hexaploid accession AUS33412, A) Original image file, B) image after color threshold to measure individual grains C) outlines created by ImageJ after measuring horizontal shape descriptors. Lower row: Vertical images of synthetic hexaploid accession AUS33412, D) Original image file, E) image after color threshold to measure individual grains F) outlines created by ImageJ after measuring horizontal shape descriptors. [file 1471-2229-14-128-S8.docx]

**A) B) C)**


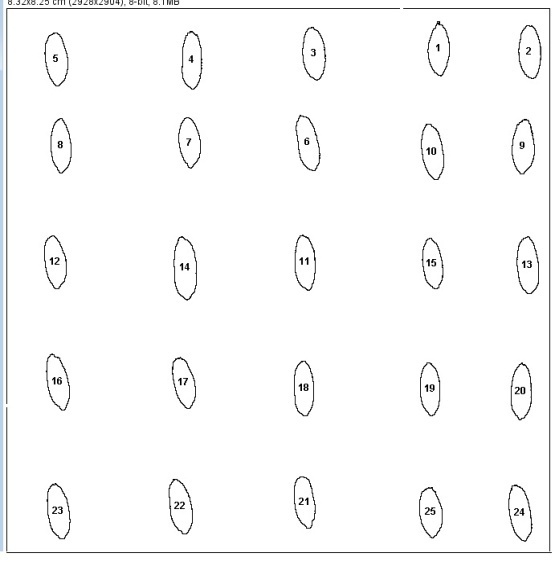
**
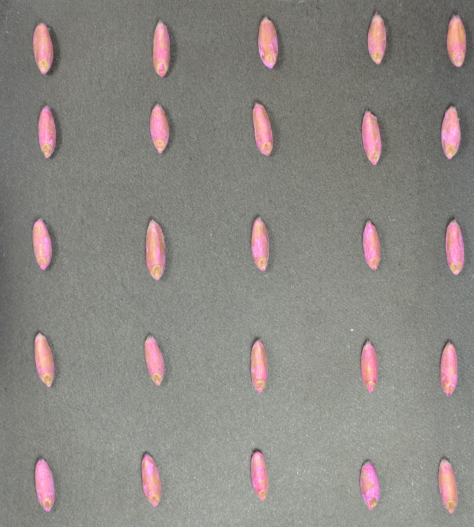

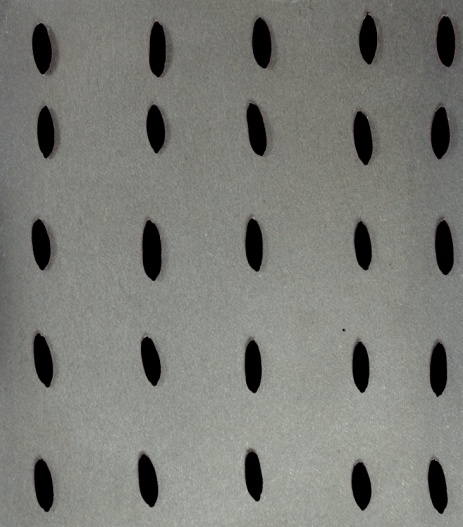
**

**D) E) F)**


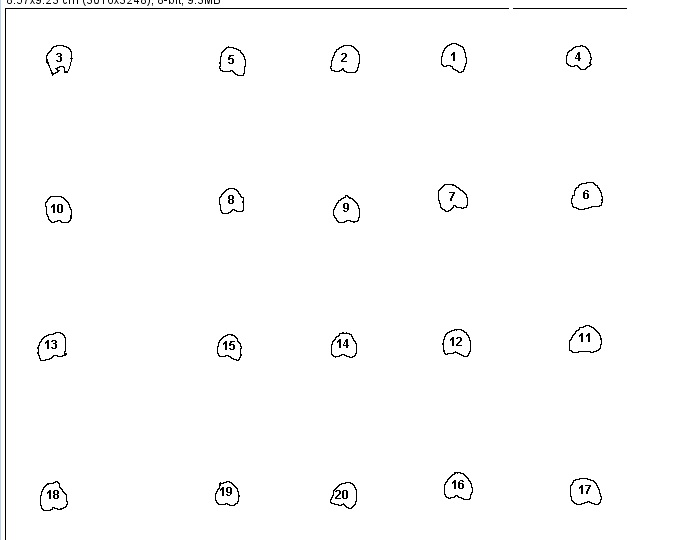
**
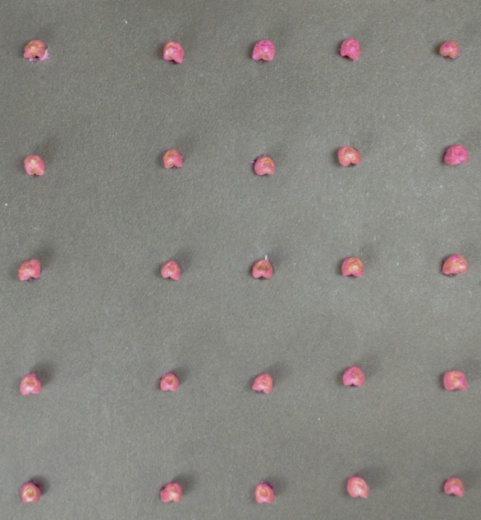

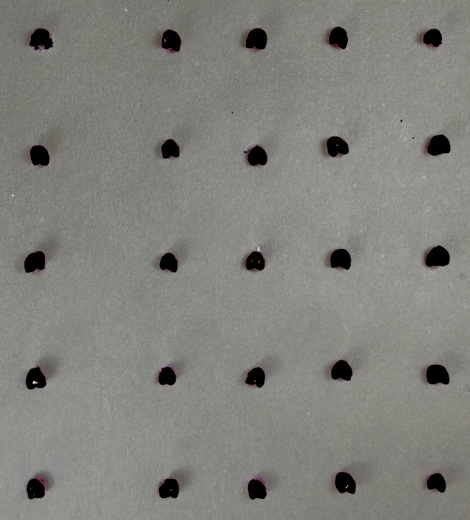
**

**Figure S2.** **Upper row:** **Horizontal** images of synthetic hexaploid accession AUS33412, **A)** Original image file, **B)** image after color threshold to measure individual grains **C)** outlines created by ImageJ after measuring horizontal shape descriptors. **Lower row:** **Vertical** images of synthetic hexaploid accession AUS33412, **D)** Original image file, **E)** image after color threshold to measure individual grains **F)** outlines created by ImageJ after measuring horizontal shape descriptors.
