# Supplementary material for: Genome-wide association for grain morphology in synthetic hexaploid wheats using digital imaging analysis
Source: BMC Plant Biol. 2014 May 9;14:128. doi: 10.1186/1471-2229-14-128 (PMC4057600; doi:10.1186/1471-2229-14-128)
Supplement: Additional file 9: Figure S3 — Dimension axis and their measurement demonstrated by ImageJ software for original grain image and its fitted ellipse. [file 1471-2229-14-128-S9.docx]

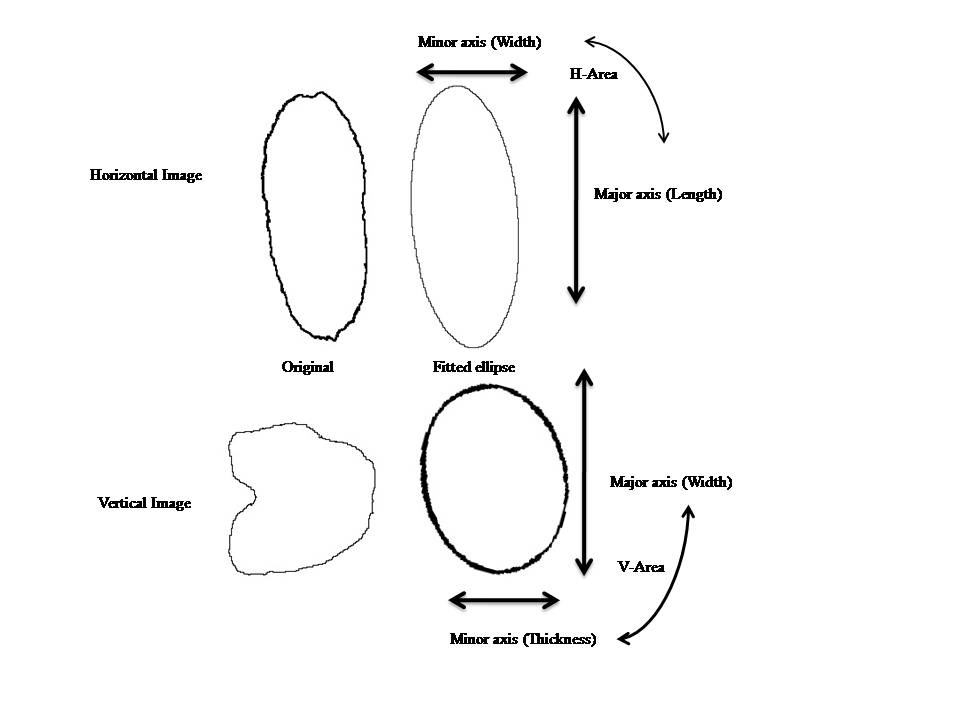


**Figure S3.** Dimension axis and their measurement demonstrated by ImageJ software for original grain image and its fitted ellipse.
