# Supplementary material for: Genome-wide association for grain morphology in synthetic hexaploid wheats using digital imaging analysis
Source: BMC Plant Biol. 2014 May 9;14:128. doi: 10.1186/1471-2229-14-128 (PMC4057600; doi:10.1186/1471-2229-14-128)
Supplement: Additional file 10: Table S3 — Photometric measurements to phenotype seed design for association genetic analysis. [file 1471-2229-14-128-S10.docx]

**Table S3.** Photometric measurements to phenotype seed design for association genetic analysis

| **Photometric measurements** | | **Abbreviation** | | **Formula for derivation** | |
| --- | --- | --- | --- | --- | --- |
| Aspect ratio | ASPECT | | $\frac{Length}{Width}$ | |  |
| Factor from density | FFD | | $Individual grain weight/(Length \times Width)$ | |  |
| Volume | VOL_XYZ_ | | $\left( \frac{4}{3} \right)\pi\left( LENGTH \right)\left( WIDTH \right)\left( THICKNESS \right)$ | |  |
| Deviation from optimal ellipse, Horizontal | PDEVH | | $PDEVH=│(p-HPERIM/HPERIM│$  where  $\dot{\begin{aligned} p=\pi[3\left( LENGTH+WIDTH \right)- \\ \sqrt{\left( 3\times LENGTH+WIDTH \right)\times(LENGTH+3\times WIDTH)} \end{aligned}}$ | |  |
| Deviation from optimal ellipse, Vertical | PDEVV | | $PDEVV=│(p-VPERIM/VPERIM│$  where  $\dot{\begin{aligned} p=\pi[3\left( THICK+WIDTH \right)- \\ \sqrt{\left( 3\times THICK+WIDTH \right)\times(THICK+3\times WIDTH)} \end{aligned}}$ | |  |
| Composite 1 | COMP1 | | (VOL_XYZ_  ⁄ PDEVH) ⁄ (PDEVV) | |  |
| Composite 2a | COMP2a | | (VOL_XYZ_) × (HPC1) × (VPC2) | |  |
| Composite 2b | COMP2b | | (VOL_XYZ_) × (HPC1) × (VPC3) | |  |
| Composite 2c | COMP2c | | (VOL_XYZ_) × (HPC1) × (VPC4) | |  |
