# Supplementary material for: Genome-wide association for grain morphology in synthetic hexaploid wheats using digital imaging analysis
Source: BMC Plant Biol. 2014 May 9;14:128. doi: 10.1186/1471-2229-14-128 (PMC4057600; doi:10.1186/1471-2229-14-128)
Supplement: Additional file 11: Figure S4 — Transformation of grain shape into five principal components to generate high-throughput quantitative data suitable for genetic analysis (Accession: AUS34404). [file 1471-2229-14-128-S11.docx]

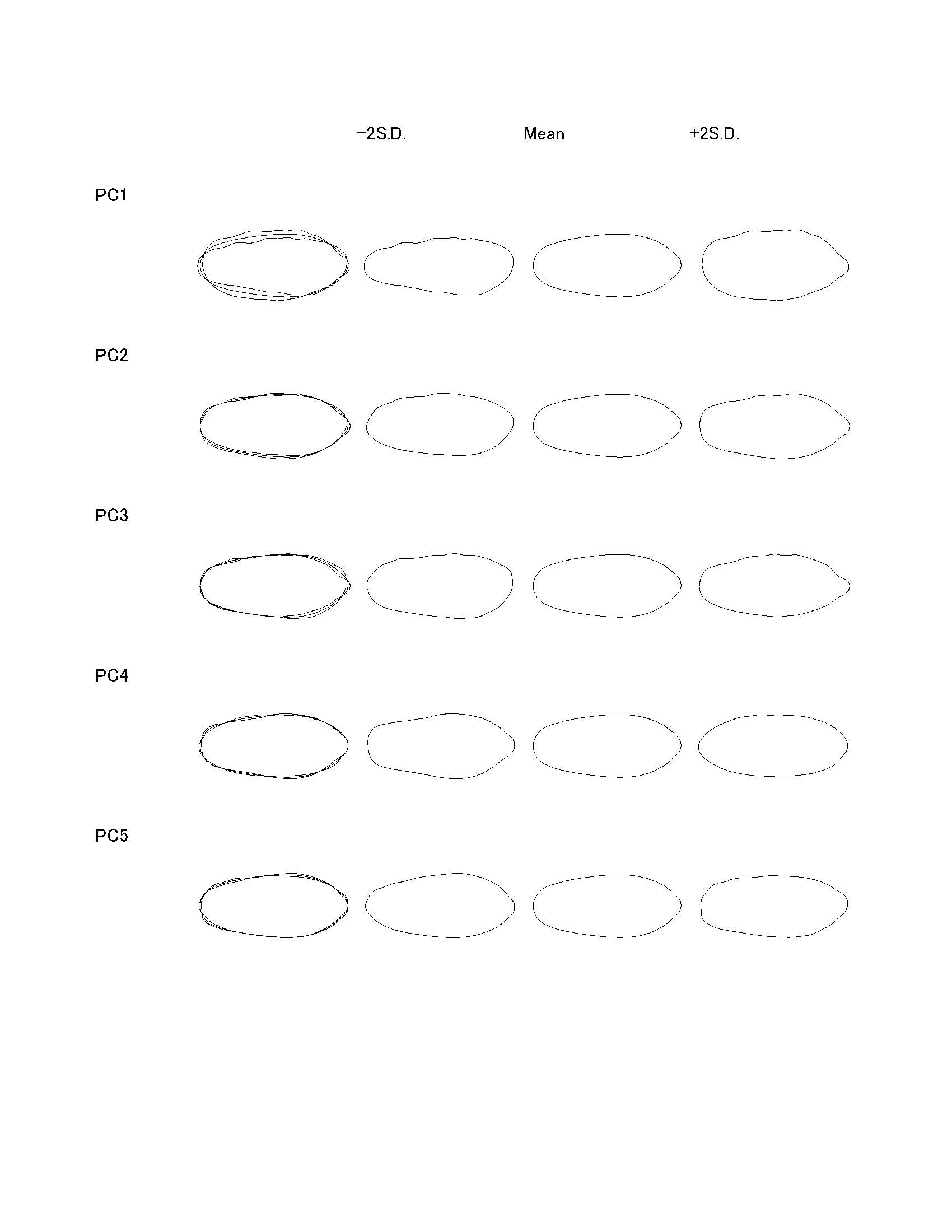

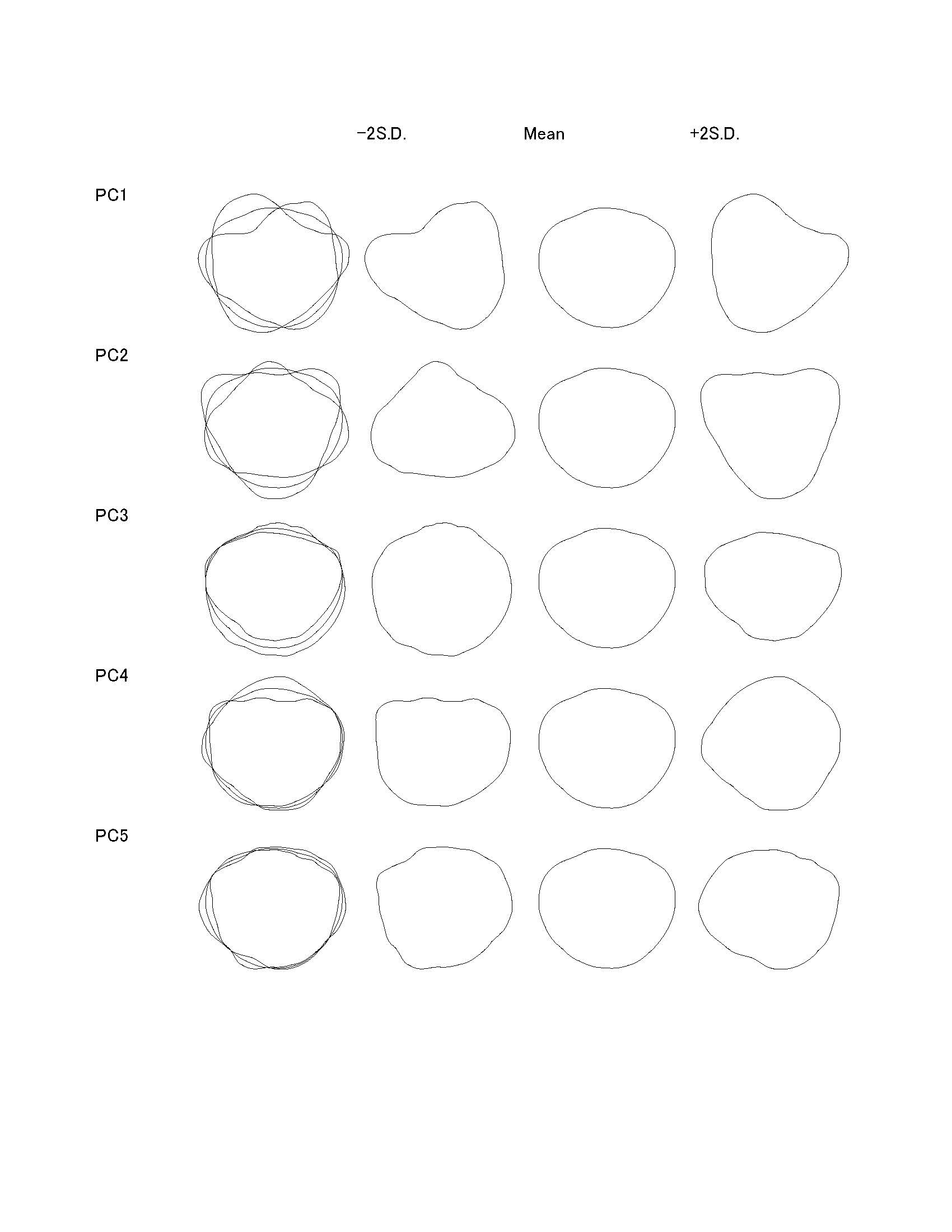


**Figure S4.** Transformation of grain shape into five principal components to generate high-throughput quantitative data suitable for genetic analysis (Accession: AUS34404)
